# The role of membrane destabilisation and protein dynamics in BAM catalysed OMP folding

**DOI:** 10.1038/s41467-021-24432-x

**Published:** 2021-07-07

**Authors:** Paul White, Samuel F. Haysom, Matthew G. Iadanza, Anna J. Higgins, Jonathan M. Machin, James M. Whitehouse, Jim E. Horne, Bob Schiffrin, Charlotte Carpenter-Platt, Antonio N. Calabrese, Kelly M. Storek, Steven T. Rutherford, David J. Brockwell, Neil A. Ranson, Sheena E. Radford

**Affiliations:** 1 Astbury Centre for Structural Molecular Biology, School of Molecular and Cellular Biology, Faculty of Biological Sciences, University of Leeds, Leeds, UK; 2 Department of Infectious Diseases, Genentech Inc., South San Francisco, CA USA; 3 Present Address: GlaxoSmithKline R&D, Stevenage, UK; 4 Present Address: Scientific Computing Department, Science and Technology Facilities Council, Didcot, UK; 5 Present Address: Department of Biochemistry, University of Oxford, Oxford, UK

**Keywords:** Protein folding, Membrane structure and assembly, Cellular microbiology, Cryoelectron microscopy

## Abstract

The folding of β-barrel outer membrane proteins (OMPs) in Gram-negative bacteria is catalysed by the β-barrel assembly machinery (BAM). How lateral opening in the β-barrel of the major subunit BamA assists in OMP folding, and the contribution of membrane disruption to BAM catalysis remain unresolved. Here, we use an anti-BamA monoclonal antibody fragment (Fab1) and two disulphide-crosslinked BAM variants (lid-locked (LL), and POTRA-5-locked (P5L)) to dissect these roles. Despite being lethal in vivo, we show that all complexes catalyse folding in vitro, albeit less efficiently than wild-type BAM. CryoEM reveals that while Fab1 and BAM-P5L trap an open-barrel state, BAM-LL contains a mixture of closed and contorted, partially-open structures. Finally, all three complexes globally destabilise the lipid bilayer, while BamA does not, revealing that the BAM lipoproteins are required for this function. Together the results provide insights into the role of BAM structure and lipid dynamics in OMP folding.

## Abstract (web summary)

The folding of outer membrane proteins (OMPs) is catalyzed by the βbarrel assembly machinery (BAM). Here, structural and functional analyses of BAM stabilized in distinct conformations elucidate the roles of lateral gate opening and interactions of BAM with the lipid bilayer in OMP assembly.

## Introduction

Outer membrane proteins (OMPs) in Gram-negative bacteria are functionally diverse but share a common β-barrel fold involving between 8 and 36 β-strands^1^. The folding and membrane insertion of OMPs is catalysed by the essential β-barrel assembly machinery (BAM)^2–4^ which in *E. coli* comprises five proteins (BamABCDE). The major conserved subunit, BamA, is a 16-stranded Omp85 family member that contains five N-terminal polypeptide transport-associated (POTRA) domains that extend into the periplasm to scaffold four lipoproteins BamB–E^5–8^, all of which are required for maximally efficient OMP folding^9,10^. BAM is essential for bacterial survival, highly conserved, and surface accessible via the extracellular loops of BamA, making the complex an attractive target for small molecule^11–13^, peptide^14,15^ and antibody-based antibiotics^16,17^.

BAM exists in an ensemble of conformations, with one of the most notable differences between published structures occurring around the seam or ‘lateral gate’ involving β-strands 1 (β1) and 16 (β16) in the BamA barrel^6–8,18–20^. In the ‘lateral-open’ conformation, as captured by the cryoEM structure of the intact complex^8^ and X-ray crystallography of the BamACDE sub-complex^5,6^, β1 and β16 are separated. In contrast, crystal structures of the intact BAM complex are in a ‘lateral-closed’ conformation both in the absence^6,7^ or presence of peptide fragments of substrate^21,22^, wherein β1 and β16 are hydrogen-bonded, albeit with fewer hydrogen bonds than exist between the other strands in the barrel^1^. The POTRA domains are also dynamic, with motions of POTRA-5 also occurring alongside changes in gate conformation, with POTRA-5 plugging the entrance to the BamA β-barrel lumen in the lateral-open structures, but moving aside when the lateral gate is closed^18^. These conformational changes are thought to be essential for cell viability as disulfide bonds that purportedly lock BamA in either conformation have a lethal phenotype that is rescued by reducing agent^6,19^. Such variants include those designed to lock the lateral gate closed (e.g. G433C/N805C linking β1 to β16^8,19^, or E435C/S665C covalently linking extracellular loop 1 (eL1) to eL6^6,19^), or to restrain the protein in an open conformation (e.g. G393C/G584C which introduces a disulfide bond between POTRA-5 and the β-turn between β8 and β9 at the base of the barrel^6^). Disulfide bonds that restrict flexibility between POTRA domains 2 and 3 also impair growth^23^; but how, or if, these motions correlate with structural changes at the BamA β-barrel is unclear.

Models of BAM-catalysed OMP insertion and folding broadly invoke two distinct roles for BAM (reviewed in ref. ^24^). Firstly conformational changes in BAM, and protein–protein interactions between BAM and substrate OMPs are thought to be involved in catalysing folding^25–29^. These models all involve a folding intermediate in which the C-terminal β-strand of the substrate is associated with BamA-β1, as supported by crosslinking^26,27^, a recent cryoEM structure of a hybrid barrel formed between BAM and tBamA (the transmembrane domain of a BamA substrate)^29^, and crystal structures of BAM covalently tethered to the C-terminal β-strands of OMP substrates OmpA and OmpLA^22^. Variations of these models include the ‘barrel elongation’^25^ and ‘swing’^27^ models which suggest that folding begins in the periplasm, and also ‘budding’ models^1,3,25^ wherein OMPs are thought to enter the lumen of the BamA barrel and fold via sequential addition of β-hairpin units^26^. This is akin to the role proposed for the mitochondrial homologue Sam50 of the sorting and assembly machinery (SAM) complex^26^. An alternative model proposes that BAM may disorder its lipid environment, lowering the kinetic barrier to OMP folding, potentially allowing OMPs to fold and insert into the outer membrane without direct interaction with the β1–β16 seam. This ‘BamA-assisted’ model^18,30–32^ is supported by molecular dynamics (MD) simulations which show lipid disordering and bilayer thinning by BamA^20,25,30–35^, and by BAM-mediated distortion of a nanodisc^18^. Both protein dynamics and lipid disordering may act synergistically to maximise the efficiency of OMP folding, and different OMPs may depend on each effect to different degrees. However, little mechanistic insight is available, beyond that which has been inferred from the observation of a lethal phenotype.

Here, we investigate the roles of BAM structure/dynamics and membrane stability in OMP folding by exploiting two disulfide-locked variants termed lid-lock (LL) and POTRA-5-lock (P5L) which are lethal in vivo^6,19^, and purportedly restrain BamA in a lateral-closed or lateral-open conformation, respectively. We also investigate a bactericidal Fab fragment (Fab1), that binds to eL4 of BamA^16^. We report cryoEM structures for the two disulphide-containing BAM variants and the BAM–Fab1 complex, revealing that BAM-P5L and Fab1 stabilise a lateral-open conformation, whilst BAM-LL adopts both a lateral-closed state and a distorted, partially open conformation. Despite being lethal in vivo, the two disulfide variants and the Fab1–BAM complex are all able to catalyse the folding of the 8-stranded OMPs OmpX and tOmpA (the transmembrane region of OmpA) in vitro, although less efficiently than wild-type BAM, and by combining Fab1 and disulfide-locking, BAM is further inactivated. We also demonstrate that all BAM variants studied lower the phase transition temperature of their lipid environment, but that BamA alone does not, providing direct experimental evidence that lipid disordering by BAM requires the presence of its lipoproteins. The results provide insights into the structural features of BAM’s catalytic mechanism and suggest that even subtle disruption of BAM activity may provide an effective route to the development of antibiotics.

## Results

### Disulfide-locked and Fab1-bound BAM catalyse OMP folding in vitro

**Fig. 1:**
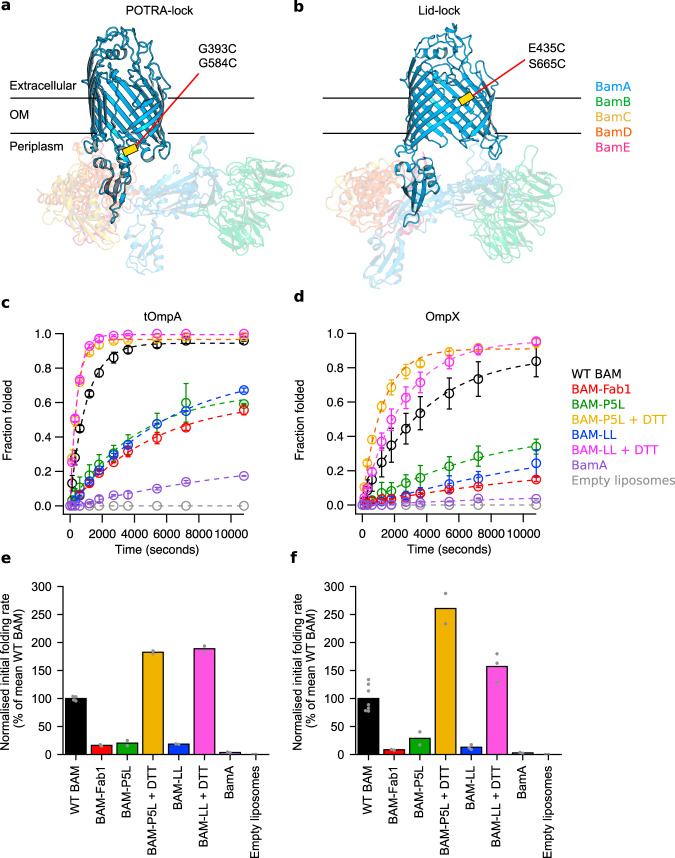
Disulfide-locked BamA variants and Fab1 binding impair BAM-mediated OMP folding in vitro. **a** BAM-P5L (G393C/G584C) is expected to lock BamA in the lateral-open conformation (PDB code 5LJO^8^), while **b** BAM-LL (E435C/S665C) is expected to lock BamA in the lateral-closed conformation (PDB code 5D0O^6^). BamA POTRAs 1–4 and BamBCDE are rendered semi-transparent for emphasis on the BamA β-barrel and POTRA-5. The position of the disulfide bond is shown as a yellow bar. Figure made in PyMOL v1.7.2.3. **c** and **d** Quantification of folded and unfolded bands from SDS–PAGE band-shift assays (Supplementary Figs. 5 and 6) plotted as fraction folded against time for tOmpA or OmpX, respectively. Data markers represent the average folded fractions calculated from at least two repeats (the number of replicates is shown in Supplementary Table 1) and dashed lines are single exponential fits of the data. Error bars represent range of values covered by the replicates. **e** and **f** The initial rates of folding (determined by applying a linear fit to the first 5% of folding data) normalised as a percentage of the mean initial rate obtained for WT BAM, are shown for **e** tOmpA and **f** OmpX folding. Bars represent the mean value for each condition, with values for each replicate shown as grey points. Average initial rates, normalised data and ranges are listed in Supplementary Table 1. Folding yields after 24 h are reported in Supplementary Table 2. Figures labelled with “BAM” refer to the full BAM complex (BamABCDE), whilst “BamA” is just BamA alone. Source data for **c**–**f** are provided as a source data file.

To assess the relationship between bacterial lethality and the catalytic ability of BAM we determined the in vitro folding activity of two paired cysteine mutations in BamA that are bactericidal^6,19^. In the BAM-P5L variant (BamA G393C/G584C)^6^, tethering of POTRA-5 to the base of the BamA barrel is expected to stabilise a lateral-open conformation (Fig. 1a). By contrast, the BAM-LL variant, (BamA E435C/S665C)^19^ is expected to lock eL1 to eL6, and stabilise a lateral-closed conformation (Fig. 1b). The BAM-LL and BAM-P5L variants were made in a BAM construct in which the two Cys of BamA that naturally form a disulfide bond (C690 and C700), are replaced with Ser (Cys-free BAM). This variant is able to complement WT BamA in *E. coli*^19,36^ and has little effect on BAM-catalysed OMP folding rates in vitro^8^. We also investigated how a bactericidal BamA-binding antibody Fab fragment, known as Fab1^16,37^, affects OMP folding in vitro. BAM-P5L, BAM-LL and the BAM–Fab1 complex were each reconstituted into liposomes comprised of *E. coli* polar lipids, and their ability to fold the 8-stranded OMPs, OmpX and tOmpA, in the presence of SurA was determined by SDS–PAGE band-shift assays^38^. In each case, BamA was folded (as judged by a band-shift relative to the boiled (denatured) BamA band) and all four BAM lipoproteins were present (Supplementary Fig. 1). Interestingly, Fab1 formed a stable, SDS-resistant complex with BamA (Supplementary Fig. 1b), consistent with its IC_50_ of 0.095 nM determined for Δ*waaD E. coli*^16^. To rule out differences in folding activity due to the size, and therefore curvature, of the liposomes, or differences in orientation of BAM within the liposomes, we determined the hydrodynamic radius (*r*_H_) of the different proteoliposomes using dynamic light scattering (DLS) and protease accessibility, respectively. DLS revealed a similar (but not identical) average *r*_H_ of the proteoliposomes ranging between 90 and 123 nm (Supplementary Fig. 2). The orientation of BAM within the liposome was also similar for all variants, with ~50% BAM/BamA in the substrate-accessible orientation (POTRA domains and lipoproteins exposed on the proteoliposome exterior) (Supplementary Fig. 3). Disulfide bond formation in BAM-P5L and BAM-LL was confirmed by the lack of fluorescein-C5-maleimide labelling (which suggested complete disulfide formation by gel densitometry and ESI-MS), and electrophoretic band-shifts in oxidising/reducing conditions (Supplementary Fig. 4). tOmpA and OmpX do not fold spontaneously into the liposomes formed from *E. coli* polar lipids, but fold rapidly and efficiently into liposomes formed from the same lipids containing WT BAM (Fig. 1c and d). Remarkably, considering their in vivo lethality^6,16,19^, the efficiency of folding and membrane insertion of tOmpA and OmpX is reduced, but not abolished, by BAM-P5L, BAM-LL and BAM–Fab1, with folding yields of ~50–60% for tOmpA and ~15–30% for OmpX after 3 h at 25 °C (note that tOmpA folds more rapidly than OmpX with WT BAM) (Fig. 1c and d, and Supplementary Figs. 5 and 6). Relative to WT BAM, the initial rates of folding for BAM–Fab1, BAM-LL and BAM-P5L ranged from 16% to 20% for tOmpA, and 8–29% for OmpX (Fig. 1e and f, respectively, and Supplementary Table 1). When the disulfide bond in BAM-P5L and BAM-LL is reduced with DTT, folding activity surpassed that of WT BAM. This difference could reflect the slightly greater proportion (~20%) of BAM-P5L and BAM-LL in the substrate accessible orientation relative to WT BAM in the proteoliposomes (Supplementary Fig. 3) (note that addition of DTT had no observable effect on folding rates for WT BAM or Cys-free BAM (Supplementary Fig. 7)). Folding into proteoliposomes containing BamA alone was much slower than observed with BAM-P5L, BAM-LL, or BAM–Fab1, with initial folding rates for both substrates reaching ~3% of that of WT BAM, highlighting the importance of the accessory lipoproteins for efficient catalysis of folding of these OMPs^39^. Importantly, the inhibited BAM variants were able to fold their OMP substrates to 84–100% completion after 24 h, whilst incubation with BamA alone resulted in folding yields of only 50% and 14% for tOmpA and OmpX, respectively, after 24 h (note that both substrates were unable to fold into empty liposomes even on these extended timescales) (Supplementary Table 2). Collectively, these results show that although both Fab1 binding and disulphide-locking of BamA are lethal in vivo^6,16,19^, the BAM-catalysed folding of OmpX and tOmpA is only partially inhibited in vitro.

### Lid-locked BAM exists in two conformations

**Fig. 2:**
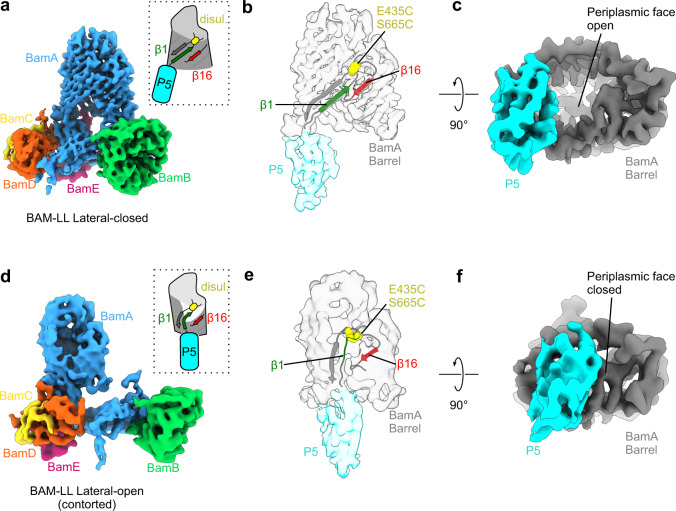
CryoEM resolves two conformations of BAM-LL in detergent. **a** 4.1 Å cryoEM map of the BAM-LL lateral-closed conformation at a contour of 10*σ*, coloured by subunit. The lateral gate is closed and POTRA-5 does not block the BamA barrel (*schematic inset*). **b** Cartoon representation of the corresponding atomic model at the lateral gate, superimposed on the segmented density for the barrel and POTRA-5 of BamA. β1 and β16 contact to close the gate. **c** The same density viewed from the periplasmic side, showing the open lumen of the BamA barrel in this conformation. **d** 4.8 Å cryoEM map of the BAM-LL lateral-open (contorted) conformation at a contour of 10*σ*, coloured by subunit. The lateral gate is open and POTRA-5 occludes the BamA barrel (*schematic inset*). **e** Cartoon representation of the corresponding atomic model at the lateral gate, superimposed on segmented density for the barrel and POTRA-5 of BamA. To satisfy the disulphide in this conformation, eL1 must bend back into the barrel to contact eL6. **f** The same density viewed from the periplasmic side, showing that the BamA lumen is blocked by POTRA-5 in this conformation. Figure made in UCSF ChimeraX^76^. Segmenting and colouring performed with corresponding atomic models. Less well-resolved regions and the micelle have been masked.

To understand the molecular basis of inhibition, we determined the structure of BAM-LL in DDM detergent micelles using cryoEM. We predicted, based on the lethality of this mutation and the crystal/cryoEM structures of BAM in its different conformational states^5–8^, that the formation of a disulfide bond between C435 and C665 would trap BAM in a lateral-closed state (Fig. 1b). However, 3D classification of cryoEM data of this construct revealed two distinct, approximately equally populated, structures (Fig. 2 and Supplementary Fig. 8). The first structure (at 4.1 Å resolution) is similar to the crystal structure of intact BAM in the lateral-closed conformation, with pairing of β1 and β16 (Fig. 2a, b) and displacement of POTRA-5 from beneath the barrel (Fig. 2c). The second structure (at 4.8 Å) has β1 and β16 separated (Fig. 2d, e) and POTRA-5 occludes the periplasmic face of the BamA barrel (Fig. 2f), and is thus consistent with a lateral-open conformation. Note that the opening/closing of the lateral gate is also accompanied by a narrowing and change in the shape of the BamA barrel, and dramatic alterations in the positioning of POTRA domains 1–4, all of which could be distinguished at the resolution of the EM maps described here. Hence we assigned the structures as lateral-open or lateral-closed conformations based on consideration of all of these criteria^18^. In all previous lateral-open structures^5,6,8^, extracellular loop 1 (eL1) bends away from the BamA β-barrel, separating the LL cysteine positions (C435 and C665) by ~20 Å. Given the unequivocal in vitro biochemical evidence for the formation of the LL disulfide (Supplementary Fig. 4), eL1 must be contorted to allow disulfide bond formation with eL6. However, poor resolution in this region of the map, itself indicative of mobility, prevented modelling of this eL1 conformation. We therefore used MD-based flexible fitting (MDFF)^40^ to morph the lateral-closed BAM-LL atomic model into the density observed in the second conformation, whilst maintaining the disulfide link. This generated a chemically plausible loop conformation (Fig. 2e), but this is not constrained by the EM density. The difference between eL1 conformations in the two BAM-LL structures is striking, and suggests that this region must be highly malleable to allow disulfide bond formation within the BamA β-barrel. Interestingly, the ‘contorted open’ BAM-LL structure closely resembles a recent structure of WT BAM in saposin nanodiscs^22^ in which eL1 adopts this inward conformation in the absence of disulfide tethering. In accord with this idea, eL1 can adopt a wide range of conformations in lateral-open BAM structures (Supplementary Fig. 9). In contrast to WT BAM^8^, density was poorly resolved for much of eL6 probably as a result of higher mobility due to the removal of the natural disulfide bond between C690 and C700. We do not believe this to be a major issue since removal of the eL6 disulfide bond did not affect activity in in vitro-folding assays (Supplementary Fig. 7). Overall, these data suggest that the LL disulfide biases the conformational ensemble toward a lateral-closed conformation, but cannot completely pull the conformational equilibrium over to that state, consistent with BAM adopting only the lateral-open state in DDM detergent^8^.

### Fab1-bound BAM and BAM-P5L adopt a lateral-open state

**Fig. 3:**
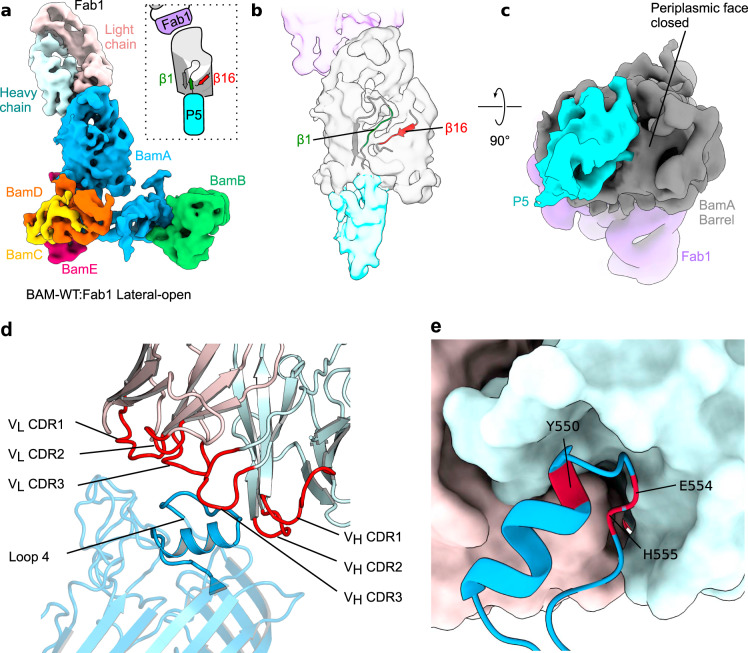
Fab1-bound BAM is in a lateral-open conformation. **a** 5.1 Å cryoEM map of the BAM–Fab1 complex in a lateral-open conformation at a contour of 10*σ*, coloured by subunit. The lateral gate is fully open and POTRA-5 occludes the BamA barrel (*schematic inset*). **b** Cartoon representation of the corresponding atomic model at the lateral gate superimposed on the segmented density for the barrel and POTRA-5 of BamA. β1 is in a conformation that makes limited contact with β16. **c** The same density viewed from the periplasmic side, showing that the BamA lumen is blocked by POTRA-5 in this conformation. Panels made using UCSF ChimeraX^76^. Segmenting and colouring performed with corresponding atomic models. Less well-resolved regions and the micelle have been masked. **d** Close up of the BamA–Fab1 interface region highlighting the Fab1 CDRs (*red*) interacting with eL4 of BamA (*dark blue)*. Other regions of BamA are rendered semi-transparent to highlight eL4. Heavy and light chains of Fab1 are coloured *cyan* and *pink*, respectively. **e** The V_L_ and V_H_ domains of Fab1 variable form a complementary binding surface for eL4 of BamA involving residues Y550, E554 and H555 (red). Sidechains for Y550, E554 and H555 are not shown as they are not well-defined in the electron density, but the close location of these residues in the binding pocket is consistent with previous mutagenesis studies^16^.

Inspired by the findings that MAB1 (and Fab1) binding is lethal in vivo^16^ and also retards OMP folding rates in vitro (Fig. 1), we next investigated the effect of Fab1 binding on the conformation of BAM using cryoEM. The structure of BAM in complex with a bactericidal molecule (Fab1) was solved in DDM micelles to 5.2 Å resolution. The cryoEM map contained unambiguous density for Fab1 bound to the extracellular region of BamA (Fig. 3a, Supplementary Fig. 10), and revealed that BAM is in a lateral-open conformation when bound to Fab1, as defined by the position of POTRA-5, the shape of the BamA β-barrel, and the orientation of β1 and β16 (Fig. 3b and c). The structure of Fab1 alone was also solved by X-ray diffraction to ~3.0 Å resolution and this structure was flexibly fitted into the EM density map (Supplementary Table 3). In agreement with mutagenesis data^16^, Fab1 binds specifically to eL4 (Fig. 3d) (contributing 98% of the total interface area of 934 Å^2^ as determined by PISA interface analysis^41^), and the complementarity determining regions (CDRs) bind to residues Y550, E554 and H555 in BamA (Fig. 3e). Interestingly, a BamA-specific nanobody (nanoE6) has also been found to bind eL4 (involving E554) and also influences dynamics in the lateral gate^17^. However, since binding of Fab1 to BAM (and nanoE6 to BamA^17^) does not drastically alter the conformation of eL4 from that seen in lateral-closed structures, how Fab1 binding stabilises a lateral-open conformation remains obscure. Finally, we determined the cryoEM structure of BAM-P5L at lower resolution (10.3 Å; Supplementary Figs. 11 and 12), and although the conformation of the lateral gate is not clearly observed at this resolution (Supplementary Fig. 12a), POTRA-5 unambiguously occludes the BamA barrel suggesting that BAM-P5L is in a “lateral-open”-like state (Supplementary Fig. 12b). Cross-correlation of the BAM-P5L, WT BAM^8^ (open) and BAM-LL (closed) density maps, as well as comparison of the shapes of the BamA barrel in the different structures, add further evidence that BAM-P5L is indeed in a lateral-open state, as expected from the design of the Cys mutants, (Supplementary Fig. 12d, e). Akin to BAM-LL, eL6 was poorly resolved in the BAM-P5L structure probably as a result of the removal of the eL6 disulfide bond.

### Fab1 binding to disulphide-locked BAM further inhibits OMP folding

**Fig. 4:**
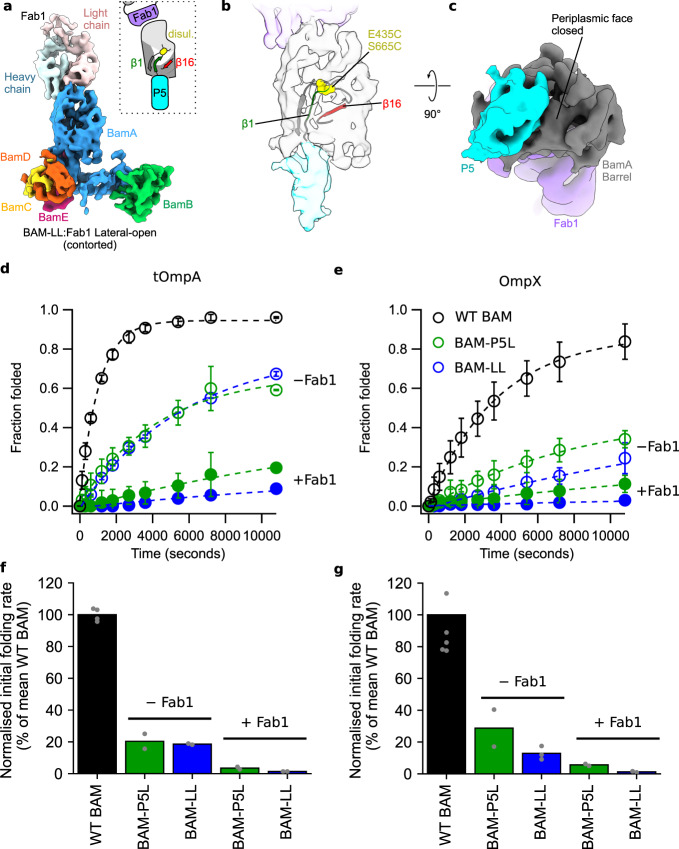
Additive effect of BAM inhibition by disulfide-locking and binding of Fab1. **a** 7.1 Å cryoEM map of the Fab1-bound LL-BAM in a lateral-open (contorted) conformation at a contour of 9.5*σ*, coloured by subunit. The lateral gate is open and POTRA-5 occludes the BamA barrel (*schematic inset*). **b** Cartoon representation of the corresponding atomic model at the lateral gate, superimposed on the segmented density for the β-barrel and POTRA-5 of BamA. To satisfy the disulphide in this conformation, eL1 must bend back into the barrel to contact eL6. **c** The same density viewed from the periplasmic side, showing that the BamA lumen is blocked by POTRA-5 in this conformation. Structural panels made using UCSF ChimeraX^76^. Segmenting and colouring performed with corresponding atomic models. Less well-resolved regions and the micelle have been masked. **d** and **e** Quantification of SDS–PAGE band-shift assays shown in Supplementary Fig. 15 for **d** tOmpA and **e** OmpX folding catalysed by BAM-P5L + Fab1 (*green, solid circles*) and BAM-LL + Fab1 (*blue, solid circles*). Data for WT BAM (*black, open circles)*, BAM-P5L (*green, open circles*) and BAM-LL (*blue, open circles*), from Fig. 1, are shown for comparison. Data markers represent the average folded fractions calculated from at least two repeats (The number of replicates is shown in Supplementary Table 1) and dashed lines are single exponential fits of the data. Error bars represent range of values covered by the replicates. **f** and **g** The initial rates, calculated by applying a linear fit to the first 5% of fitted folding data, were normalised to the mean value for WT BAM, and are shown for **f** tOmpA and **g** OmpX folding. Bars represent the mean value for each condition, with values for each replicate shown as grey points. Average initial rates, normalised data and ranges are listed Supplementary Table 1. Folding yields after 24 h are reported in Supplementary Table 2. Figures labelled with “BAM” refer to the full BAM complex (BamABCDE), whilst “BamA” is just BamA alone. Source data for **d**–**g** are provided as a source data file.

As BAM can populate a lateral-open conformation in the presence or absence of Fab1, we determined the cryoEM structure of BAM-LL bound to Fab1 to ascertain whether Fab1 binding could further stabilise a lateral-open conformation, potentially further blocking the conformational changes required for BAM’s catalytic action. In contrast with BAM-LL, the cryoEM structure of the BAM-LL:Fab1 complex (at 7.1 Å resolution) contains a single structure that is in a lateral-open (contorted) conformation (Fig. 4a, Supplementary Fig. 13), consistent with Fab1 biasing BamA’s conformational equilibrium towards a lateral-open state (Fig. 4b) in which POTRA-5 occludes the barrel (Fig. 4c). Further evidence for the lateral-closed state being incompatible with Fab1 binding was observed by SDS–PAGE, where the SDS-resistant BamA-Fab1 band observed for WT BAM-Fab1 was weaker for BAM-LL-Fab1, with a compensating increase in the band corresponding to non-complexed BamA, suggestive of the BAM-LL:Fab1 complex being less stable under SDS–PAGE conditions (Supplementary Fig. 14a). Interestingly, since MAB1 binds to BAM in the OM of ΔwaaD *E. coli*^16^, this suggests that a lateral-open conformation is formed in situ in the OM, consistent with previous data^36^. Conversely, the Fab1-bound BAM-P5L complex produces an SDS-resistant band, consistent with stable binding to its lateral-open state (Supplementary Fig. 14b). tOmpA and OmpX folding assays revealed that the addition of Fab1 to BAM-P5L or BAM-LL each resulted in increased inhibition, with folding yields of ~10–20% for tOmpA (Fig. 4d, Supplementary Fig. 15a) and 5–10% for OmpX (Fig. 4e, Supplementary Fig. 15b) after 3 h at 25 °C, and initial folding rates of only 1–4% and 1–6% of that of WT BAM for tOmpA and OmpX, respectively (Fig. 4f and g). This additive inhibition could arise from a synergistic reduction in conformational dynamics within the BAM complex, or from Fab1 binding and disulphide locking inhibiting distinct mechanisms of BAM-mediated folding catalysis.

### BAM lipoproteins mediate destabilisation of the lipid bilayer

**Fig. 5:**
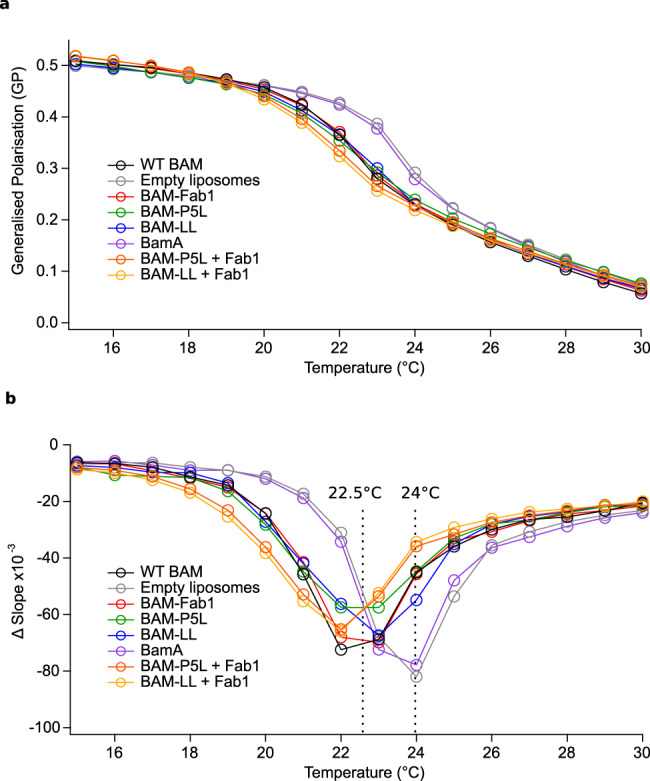
BAM variants reduce the phase transition temperature of DMPC liposomes. Global lipid phase transition behaviour for each BAM variant and BamA in DMPC proteoliposomes, with an empty liposome control measured using laurdan fluorescence. **a** The ratio of laurdan fluorescence at 440 and 490 nm was plotted as generalised polarisation (GP, see the “Methods” section) against temperature for 0.8 µM BAM/BamA proteoliposome suspensions at a 1600:1 (mol/mol) lipid-to-protein ratio (LPR) with added laurdan (at a 305:1 lipid-to-laurdan ratio) in TBS pH 8.0. **b** The first derivative of data shown in **a** showing the transition temperature for each liposome suspension as the point of steepest (most negative) gradient. Whilst empty DMPC (*grey*) and BamA proteoliposomes (*purple*) have a transition temperature of 24 °C, the presence of WT BAM (*black*), BAM-Fab1 (*red*), BAM-P5L (*green*), BAM-LL (*blue*), BAM-P5L + Fab1 *(orange)* and BAM-LL + Fab1 *(yellow)* broaden the phase transition and lower the transition temperature. Figures labelled with “BAM” refer to the full BAM complex (BamABCDE), whilst “BamA” is just BamA alone. This experiment was performed once for each variant shown. Source data for **a** and **b** are provided as a source data file.

In vitro studies have shown that spontaneous OMP-folding rates and efficiencies are increased in membranes with decreased thickness, increased fluidity, or containing bilayer defects^42–45^. As well as directly interacting with its substrate OMPs^27,29^, BAM is also thought to reduce the stability of the lipid bilayer to facilitate folding, due to asymmetry in the hydrophobic thickness of the BamA β-barrel (which is narrowest in the vicinity of the lateral gate)^18,32^. Evidence for membrane destabilisation has been provided by MD simulations of BamA in lipid bilayers^20,24,25,30–35^ and by cryoEM and MD simulations of BAM in nanodiscs formed from *E. coli* polar lipids^18^. To determine how the different conformational states of BAM affect global bilayer stability more directly, we measured the effect of the different BAM complexes studied above on the lipid phase transition of liposomes formed from 1,2-dimyristoyl-*sn*-glycero-3-phosphocholine (DMPC, *di*C_14:0_PC) using the fluorescent lipid probe laurdan, the fluorescence emission spectrum of which depends on lipid phase^46^. While DMPC is not found in the *E. coli* outer membrane, it was chosen for these experiments as it undergoes a gel–liquid phase transition with a midpoint of ~24 °C (compared with ~3 °C for *E. coli* polar lipid^47^) and BAM has been shown to be active in DMPC liposomes^48^. Having successfully produced DMPC proteoliposomes containing different variants of the BAM complex and BamA (Supplementary Fig. 16), along with empty DMPC liposomes, laurdan was introduced to partition into the bilayer and report on its stability. As expected, a phase transition for empty DMPC liposomes was observed at 24 °C (Fig. 5a, see also Supplementary Fig. 17). Interestingly, the transition phase temperature (*T*_m_) was not affected by the presence of BamA alone (Fig. 5a), demonstrating that the asymmetric BamA β-barrel does not itself cause global perturbation of the lipid bilayer, at least as judged by this assay (although local perturbation close to the BamA barrel, as reported previously by MD simulations^18,20,24,25,30–35^ cannot be ruled out). By contrast, in all proteoliposomes containing the full BAM complex, regardless of whether that complex is inhibited, the gel–liquid phase transition occurred at a lower temperature (~22–23 °C) and over a broader temperature range (Fig. 5b). These results thus demonstrate that BAM disrupts global bilayer stability independently of the structure of the β1–β16 seam and shows that one or more of the BamB–E lipoproteins are essential for this perturbation of the membrane.

## Discussion

Protein–protein interactions between BAM and substrate OMPs, and lipid disordering have both been implicated as important features in BAM function^3,24^, but how these different facets of BAM are balanced to enable OMP folding remained unclear. Here, we have used structural, biochemical and kinetic refolding analyses to dissect these two roles, at least for the 8-stranded OMPs, tOmpA and OmpX. BAM is well-known to be conformationally dynamic, with cryo-EM and X-ray structures capturing the complex in lateral-open^5,6,8^ and lateral-closed^6,7,21,22^ conformations, and a recent cryoEM, MD and single-molecule FRET study demonstrating dynamics of the complex in nanodiscs^18^. Furthermore, recent X-ray structures have demonstrated that the C-terminal strand of the OMP substrates OmpA and OmpLA forms an antiparallel β-strand pairing with lateral-closed BamA β1, possibly capturing an early stage intermediate in OMP assembly^22^. A recent cryoEM structure of a BAM:tBamA complex revealed that the tBamA substrate forms a β-strand pairing with lateral-open BamA β1 of BAM, whilst making a side-chain mediated interface involving BamA β16, to form a hybrid barrel^29^ that presumably mimics a late-stage assembly intermediate. This observation is consistent with crosslinking studies of EspP^27^ and LptD^28^ to BAM, and Por1 to SAM^26^. Given these insights, it is perhaps unsurprising that trapping BamA in the BAM complex in an open or closed conformation by disulfide bonding has a profound effect on bacterial viability, akin to the observations found using nanobodies^17^, small molecules and peptidomimetic antibiotics, which also have a lethal outcome^11,12^. Remarkably, we show here that this in vivo lethality masks a more subtle effect on BAM activity that is revealed by in vitro activity assays. We were able to show that WT BAM is capable of folding the 8-stranded OMPs tOmpA and OmpX into liposomes formed from *E. coli* polar lipid (noting that these membrane are much less complex than the crowded OM which contains lipopolysaccharide (LPS) in its outer leaflet), and no folding was observed in the absence of BAM. Both disulfide-locking and Fab1-binding inhibit, but do not abolish, BAM-catalysed folding of these substrates (Fig. 1, and Supplementary Tables 1 and 2). The finding that these inhibitory effects are distinct and additive (Fig. 4) highlights the importance of different, presumably parallel, facets of BAM action for OMP folding catalysis. We also observed that tOmpA folds faster than OmpX for all BAM variants despite having a slower intrinsic rate of folding into lipid^43^. We speculate that OmpX may fold more slowly than tOmpA in our assays as it has a higher affinity for SurA^31,49,50^ and consequently, a slower rate of delivery to BAM for folding.

Lateral-open and lateral-closed conformations and their interconversion are functionally important, with the lateral-closed conformation with an accessible β1 strand considered to be the substrate-acceptor state. Our cryoEM structures confirm that in solution, both BAM-P5L and Fab1 restrain BamA in a lateral-open conformation (Figs. 3, 4, and Supplementary Fig. 12). Presumably, this prevents substrate access and pairing to BamA β1 which recent structures suggest initially occurs to a lateral-closed conformation^22^. It may also inhibit substrate binding by occlusion of entry to the BamA barrel by POTRA-5. Consistent with this, it has recently been shown that the BAM substrate, RcsF, binds in the lumen of the BamA β-barrel only in the lateral-closed conformation^21^, and that the essential mediator of LPS assembly, LptD, contacts the internal lumen of BamA during folding^28^. An inability to assemble larger and essential BAM-dependent substrates, such as LptD, could explain why disulfide locking/Fab1 binding are lethal in vivo^6,16,19^, despite smaller OMPs potentially remaining able to fold and insert into the OM, albeit more slowly than with WT BAM. For the latter OMPs, lethality may result from a reduced flux through the OMP biogenesis pathway when BAM is impaired, inducing cell envelope stress caused by accumulation of unfolded OMPs in the periplasm. Indeed, increased envelope stress was observed upon addition of MAB1 to Δ*waaD E. coli*^16^. Moreover, a small molecule inhibitor of the regulator of sigma E protease (RseP)^51^, that is a key component of this pathway, has a lethal outcome by blocking the σ^E^ stress response that normally responds to envelope stress by increasing BAM expression^52^, decreasing OMP expression^53^, and increasing protein degradation^54^. The extent to which the folding of larger OMPs is inhibited by the BAM variants examined here remains unclear, but we speculate that for these proteins there could be a greater dependence on a direct interaction with BAM for successful insertion and folding, with BAM being unable to destabilise membranes sufficiently to allow larger OMPs to fold solely via this route.

Despite the apparent incompatibility of BAM-LL’s disulfide bond and a lateral-open conformation^6,8^, both open-like and closed structures are present in approximately equal populations in solution. The BAM-LL structures presented here thus provide direct evidence that at least β1 and β2 of BamA are malleable in the lateral-open state, being able to bend inwards towards the barrel lumen (Supplementary Fig. 9). Such plasticity appears to be functionally relevant, especially considering the more severe outward motion observed when BAM is engaged with tBamA as a substrate^29^ (Supplementary Fig. 9). Such an extended conformation would presumably be impossible in BAM-LL, perhaps explaining the partial inhibitory effects observed here for OmpX and tOmpA. Superposition of all the lateral-open BAM structures reported to date thus support a model in which the N-terminal half of the BamA barrel is conformationally dynamic, whilst the C-terminal half provides a stable scaffold that supports these functionally important conformational changes.

Lipid destabilisation by BAM has been proposed previously as a potentially important facet of the catalysis of OMP folding and insertion into the OM^3,25,55^. This has been supported by MD simulations that reveal destabilisation of the membrane surrounding BamA^20,24,25,30–35^, and a recent cryoEM structure of BAM in nanodiscs containing *E. coli* polar lipids that shows the distortion of the bilayer adjacent to the lateral gate^18^. Whilst these effects are localised to the BamA barrel, the laurdan fluorescence data provide direct biochemical evidence that BAM causes global destabilisation of a bilayer, as revealed by a reduction in the lipid phase transition temperature of DMPC liposomes (Fig. 5). They also reveal that this is mediated by one or more of the lipoproteins BamB–E, either directly, or indirectly by their effect on BamA, since BamA alone had no discernible effect. Importantly, cryoEM structures of BAM have identified interactions between BamB, BamD and BamE and detergent micelles^8^, and with lipid in nanodiscs (with POTRA-3 making additional membrane contacts in nanodiscs)^18^, and BamC is thought to span the membrane entirely^56^. It seems plausible, therefore, that these interactions, separately or in combination, could affect membrane stability. Interactions of the POTRA domains with the lipid surface have also been reported previously^57^. Local bilayer disordering effects mediated by BamA that are visualised by molecular dynamics simulations^18,20,24,25,30–35^ are presumably beyond the sensitivity of the laurdan assay, and could account for the residual folding activity of BamA and the doubly inhibited BAM variants. In addition to the roles of BamB–E in substrate recognition^15,58^, the lipoproteins mediate BAM oligomerisation into ‘precincts’^59^ and coordinate conformational changes in BamA^36,60^. Since the global liposome-disordering effect is retained in the locked BAM variants, we suggest that lipoprotein-mediated BAM oligomerisation reduces liposome stability. The results presented here thus highlight the importance of these lipoproteins in mediating changes in membrane stability, an effect that could be highly significant in the crowded OM.

In summary, the results presented allow different facets of BAM-mediated catalysis of OMP folding and membrane insertion to be discerned. By structural analysis of Fab1-bound and two different disulfide-locked BAM complexes we reveal a remarkable structural malleability of the BamA barrel, and show that interconversion between these different structures is essential for efficient folding and membrane insertion of the 8-stranded tOmpA and OmpX substrates in vitro. In addition, we provide direct biochemical evidence that BAM causes global destabilisation of a lipid bilayer and reveal that this is not endowed by asymmetry in the depth of the BamA barrel, but instead requires the presence of BamB–E, demonstrating a role for its lipoproteins in this function. Finally, by demonstrating a significant, but reduced folding capacity of the Fab1-bound and disulfide-locked BAM variants in vitro, we provide evidence in support of models that suggest that bacterial viability depends on a delicate balance between the rates of OMP synthesis and their chaperone-dependent delivery to BAM, with the catalytic power of BAM to insert OMPs into the OM. Perturbing this balance thus offers exciting opportunities to create antibacterial agents by targeting the different protein complexes required for OMP biogenesis.

## Methods

### Expression and purification of WT and disulfide-locked BAM complexes

BAM-LL (BamA(E435C/S665C/C690S/C700S)BCDE-His_6_) and BAM-P5L (BamA(G393C/G584C/C690S/C700S)BCDE-His_6_) in a pTrc99a vector were generated using Q5 site-directed mutagenesis (New England BioLabs) using plasmid pJH114 (kindly provided by Harris Bernstein^61^) as a template. WT BAM, BAM-LL and BAM-P5L were expressed in *E. coli* BL21(DE3) cells and were purified from the membrane fraction using a combination of Ni-affinity and size-exclusion chromatography^8^.

### Expression and purification of BamA, OmpX and tOmpA

BamA, OmpX and tOmpA were expressed as inclusion bodies in *E. coli* BL21(DE3) cells, using a procedure modified from McMorran et al. ^50^. Briefly, inclusion bodies were solubilised in 25 mM Tris–HCl pH 8.0, 6 M guanidine–HCl and were centrifuged (20,000×*g*, 20 min, 4 °C) to remove remaining insoluble material. The solubilised inclusion bodies were purified by SEC using a Superdex 75 HiLoad 26/60 column (GE Healthcare) for tOmpA and OmpX, and Sephacryl 200 26/60 column for BamA, equilibrated in 25 mM Tris–HCl pH 8.0, 6 M guanidine–HCl. For folding experiments, OmpX and tOmpA were buffer exchanged into Tris-buffered saline (TBS, 20 mM Tris–HCl, 150 mM NaCl) pH 8.0, 8 M urea using Zeba™ Spin Desalting Columns, 7k MWCO, 0.5 mL (Thermo Scientific). BamA was refolded in LDAO detergent prior to reconstitution into proteoliposomes, as described previously^62^ (see below).

### Refolding of BamA

BamA was refolded as described by Hartmann et al. ^62^. Briefly, BamA was added dropwise into ice-cold 50 mM Tris–HCl pH 8.0, 300 mM NaCl, 500 mM arginine, 0.5% (w/v) LDAO, 10 mM DTT whilst rapidly stirring. Following 24 h incubation, BamA was dialysed against 50 mM Tris–HCl pH 8.0, 0.1% (w/v) LDAO overnight before loading on a 5 mL HiTrap Q (GE Healthcare) anion exchange column and eluting in a NaCl gradient. Folded BamA was separated from unfolded and degraded BamA, as judged by SDS–PAGE, and used for reconstitution into liposomes containing *E. coli* polar lipid or DMPC, as required.

### Expression and purification of SurA

SurA with an N-terminal 6x His-tag and a TEV cleavage site was expressed and purified using a modified protocol described previously^50^. Briefly, SurA was expressed in *E. coli* BL21(DE3) cells and was purified on a 5 mL HisTrap FF column (GE Healthcare). SurA was denatured on-column in 25 mM Tris–HCl pH 7.2, 6 M guanidine–HCl, washed in the same buffer and then refolded on-column in 25 mM Tris–HCl pH 7.2, 150 mM NaCl, 20 mM imidazole before elution in 25 mM Tris–HCl pH 7.2, 150 mM NaCl, 500 mM imidazole. The His-tag was cleaved by addition of His-tagged TEV protease (produced as described in ref. ^31^) and 14.3 mM 2-mercaptoethanol, and the cleaved His-tag and TEV protease were removed on a 5 mL HisTrap FF column. Purified SurA was dialysed against 5 L TBS pH 8.0, concentrated to ~200 µM using Vivaspin 20 MWCO 10 kDa concentrators (Sartorius, UK), aliquoted, snap-frozen in liquid nitrogen, and stored at −80 °C.

### Monoclonal antibody Fab production

Fabs were cloned and expressed in *E. coli* as described in refs. ^63,64^. Cell paste containing the expressed Fab was resuspended in PBS buffer containing 25 mM EDTA and 1 mM PMSF. The mixture was homogenised and then passed twice through a microfluidiser. The suspension was then centrifuged at 21,500 × *g* for 60 min. The supernatant was loaded onto a Protein G column equilibrated with PBS at 5 mL/min. The column was washed with PBS to baseline and proteins were eluted with 0.6% (v/v) acetic acid. Fractions containing Fabs, assayed by SDS–PAGE, were pooled and loaded onto a 50 mL SP Sepharose column equilibrated in 20 mM MES, pH 5.5. The column was washed with 20 mM MES, pH 5.5 for 2 column volumes and the protein was then eluted with a linear gradient to 0.5 M NaCl in the same buffer. For final purification, Fab-containing fractions from the ion exchange column were concentrated and run on a Superdex 75 size-exclusion column (GE Healthcare) in PBS buffer.

### Reconstitution of BAM complex variants and BamA into *E. coli* polar lipid proteoliposomes

*E. coli* polar lipid extract, purchased as powder from Avanti Polar Lipids (Alabaster, AL), was dissolved in 80:20 (v/v) chloroform/methanol at 20 mg/mL. Appropriate volumes were dried to thin films in clean Pyrex tubes at 42 °C under N_2_ gas, and were further dried by vacuum desiccation for at least 3 h. WT BAM, BAM-LL and BAM-P5L in TBS pH 8.0, 0.05% (w/v) DDM were mixed with *E. coli* polar lipid extract films solubilized in TBS pH 8.0, 0.05% (w/v) DDM in a 1:2 (w/w) ratio. For formation of BAM-Fab1 proteoliposomes, a 2-fold molar excess of Fab1 was added to WT BAM, BAM-P5L or BAM-LL in TBS pH 8.0, 0.05% (w/v) DDM before mixing with lipid. For BamA proteoliposomes, refolded BamA was added to *E. coli* polar lipid films solubilised in TBS pH 8.0, 0.1% (w/v) LDAO in a 1:2 (w/w) ratio. Empty liposomes were prepared by mixing lipid with an equivalent volume of buffer. To remove detergent and promote liposome formation, the mixtures were dialysed against 2 L of 20 mM Tris–HCl pH 8.0, 150 mM KCl using 12–14 kDa MWCO D-Tube™ Maxi Dialyzers (Merck) at room temperature for 48 h with a total of four buffer changes. Following dialysis, the proteoliposomes were pelleted twice by ultracentrifugation at 100,000 × *g* for 30 min at 4 °C (the supernatants referred to as wash 1 and wash 2 in Supplementary figures) and were resuspended in TBS pH 8.0. Protein concentration was determined using a BCA assay (ThermoScientific) and successful reconstitution was determined by SDS–PAGE.

### Dynamic light scattering

Proteoliposomes were diluted to a lipid concentration of ~5–10 µg/mL and 300 µL was injected into a Wyatt miniDawnTreos^®^ system (equipped with an additional DLS detector). Cold, filtered (0.22 μm) TBS pH 8.0 buffer was used to obtain ~5 min baselines before and after sample injection. A 3-min sample window was used for the analysis by the software. The flow cell was flushed with 1 mL 0.22 µm filtered and degassed 1 M nitric acid and 2 mL 18 MΩ H_2_O after each run, followed by 1 mL of buffer. Correlation curves were analysed, using the Astra 6.0.3^®^ software, by regularisation^65^. All samples were measured three times.

### Trypsin proteolysis of proteoliposomes

*E. coli* polar lipid proteoliposomes containing BAM complex variants or BamA in TBS pH 8.0 were digested with addition of Porcine Sequencing-grade Modified Trypsin (Promega V5111) at a protein/trypsin ratio of 50:1 (w/w). The final BAM/BamA concentration was 2 µM and, for detergent-solubilised controls, DDM was added to the buffer at 1% (w/v) concentration. Digests and a non-digested control were incubated at 37 °C for 16 h before terminating the reaction by boiling for 10 min in SDS–PAGE loading buffer. Digests and controls were analysed on 15% (w/v) SDS–PAGE gels. The proportion of BAM complexes orientated with POTRA domains/lipoproteins on the exterior side of the liposome was determined by dividing the total band intensity corresponding to intact BamA–E for the digest reaction, by that of the non-digested control. Data are presented as a percentage. The DDM-permeabilised sample confirmed that the proteolysis reaction had reached completion.

### Fluorescein-C5-maleimide labelling of free thiols in BAM disulfide variants

BAM-LL and BAM-P5L proteoliposome preparations (containing 5 µM BAM) in TBS pH 8.0 were treated with 1 mM TCEP or 0.1 mM diamide, along with an untreated control, for 45 min at room temperature. The proteoliposomes were then diluted 10-fold into TBS pH 7.5, 8 M urea containing 100 µM fluorescein-C5-maleimide (5-MF) and were incubated overnight at 25 °C. The products of the labelling reaction were then analysed by SDS–PAGE on 15% (w/v) acrylamide/bis-acrylamide (37.5:1) Tris–tricine SDS–PAGE gels run at 60 mA per gel for 90 min at 25 °C, and imaged under 460 nm light using an Alliance Q9 Advanced gel doc (UVITEC, Cambridge, UK). Subsequently gels were stained with InstantBlue™ (Experion) to visualise all protein bands and verify equal loading. Fluorescence intensity (FI) of the BamA band for each sample was quantified using ImageJ software (Fiji), and percentage disulphide formation calculated.

### Mass spectrometry of thiol-labelled BAM-LL

Samples of purified BAM-LL (20 µM, 11.5 µL) in TBS pH 8.0, 0.05% (w/v) DDM were incubated in the presence or absence of 1 mM TCEP for 45 min at room temperature. These samples were then diluted 20-fold into TBS pH 7.5, 6 M Guanidine–HCl containing 250 µM 5-MF or N-ethyl maleimide (NEM) and labelling allowed to proceed for 1.5 h. A sample of untreated BAM was also diluted into the same buffer without label to obtain an unlabelled spectrum. 200 µL of each sample was then precipitated using chloroform–methanol to separate protein from unreacted label and other contaminants. Briefly, methanol (600 µL) and chloroform (200 µL) were added and the solution mixed by vortexing, before centrifuging (10,000 × *g*, 2 min). Water (400 μl) was then added, and the solution vortexed and centrifuged as before. The upper aqueous layer was then removed (leaving precipitated protein floating atop the lower organic phase). Methanol (400 μl) was then added, before vortexing and centrifuging as before. Finally the supernatant was removed and the protein pellet dried under N_2_ gas for 30 min.

The dried pellet was suspended in 20 µL of 20% (v/v) formic acid immediately prior to analysis by ESI-MS to avoid formylation. For analysis 2 µL sample was diluted with 8 µL water. Proteins were analysed intact by ESI mass spectrometry using online desalting liquid chromatography–MS on a nanoAcquity LC system interfaced to a Xevo G2-S mass spectrometer (Waters Ltd., Wilmslow, Manchester, UK). The sample (1 μl) was loaded onto an Acquity UPLC Protein BEH C4 column (300 Å, 1.7 µm, 2.1 mm × 100 mm, Waters UK) with an Acquity UPLC Protein BEH VanGuard Pre‐Column (300 Å, 1.7 µm, 2.1 mm × 5 mm, Waters UK). The BEH C4 column was washed with 10% (v/v) solvent B in solvent A (solvent A was 0.1% (v/v) formic acid in water, solvent B was 0.1% (v/v) formic acid in acetonitrile) for 3 min at 50 μl min^−1^. After valve switching, the bound proteins were eluted using a gradient of 10–95% (v/v) solvent B in A over 10 min at 50 μl min^−1^. The column was subsequently washed with 95% (v/v) solvent B in A for 6 min and re-equilibrated with 5% (v/v) solvent B in solvent A for the next injection. The column eluant was directed into the mass spectrometer via a Z-spray electrospray source. The MS was operated in positive TOF mode using a capillary voltage of 3.0 kV, sample cone of 60 V and source offset of 80 V. The source temperature was 100 °C and desolvation was 250 °C. Mass calibration was performed by a separate injection of 100 fmol µL^−1^ Glu-1-Fibrinopeptide B. Data processing was performed using MassLynx v4.2.

### BAM-mediated folding of OMPs by SDS–PAGE band-shift assays

Solutions of 20 µM tOmpA or OmpX denatured in TBS pH 8.0 containing 8 M urea were diluted 5-fold into a 20 µM solution of SurA. This mixture was then immediately diluted 2-fold into BAM, BamA or empty proteoliposomes to initiate the folding reaction, maintained at 25 °C. Final concentrations were 1 µM BAM, 2 µM tOmpA/OmpX, 10 µM SurA, 0.8 M urea in TBS pH 8.0. DTT was included in the relevant folding reactions at a final concentration of 25 mM. Samples of the folding reaction were taken periodically and were quenched in SDS–PAGE loading buffer (final concentrations: 50 mM Tris–HCl pH 6.8, 10% (v/v) glycerol, 1.5% (w/v) SDS, 0.001% (w/v) bromophenol blue). The samples, including a boiled control (10 mins at >95 °C), were run on 15% (w/v) SDS–PAGE gels as described above. The gels were stained in InstantBlue™ (Experion) and were imaged using an Alliance Q9 Advanced gel doc (UVITEC, Cambridge, UK). Folded and unfolded band intensities were quantified using ImageJ software (Fiji) and were plotted as a fraction folded (*I*_F_/(*I*_F_ + *I*_UF_)) against time. Folding data were fitted to a single exponential function in Igor Pro (V8.04) and initial rates calculated by applying a linear fit to data within the first 5% of the time-course (540 s).

### CryoEM grid preparation

Samples for grid preparation were prepared as follows. Purified BAM-LL or BAM-P5L in 50 mM Tris–HCl pH 8.0, 150 mM NaCl and 0.05% (w/v) DDM were diluted to 3.3 or 2.3 mg/mL, respectively. For the BAM–Fab1 complex, purified WT BAM was mixed with a 2-fold molar excess of Fab1 and run on a Superdex 200 10/300 column in TBS pH 8.0, 0.05% (w/v) DDM to isolate a stoichiometric complex from excess free Fab1. Fractions corresponding to the complex were concentrated to 4.8 µM using Vivaspin 500 concentrators MWCO 30k (Sartorius). To assemble the Fab1-bound BAM-LL complex, stock solutions of purified BAM-LL and Fab1 were first diluted to 5.9 μM in 20 mM Tris–HCl pH 8.0, 150 mM NaCl and 0.05% (w/v) DDM and mixed in a 1:1 molar ratio, before dilution in detergent-free buffer to a total protein concentration of 0.9 mg/mL and a total DDM concentration of 0.03% (w/v). The detergent concentration was lowered to combat a tendency for very thin ice on the resulting grids.

CryoEM grids were prepared as follows. For the BAM–Fab1 complex, 4 µL protein was applied to gold UltrAUfoil R2/2 200 mesh grids, previously glow discharged for 60 s at 20 mA in a GlowQube Plus (Electron Microscopy Sciences) in the presence of amylamine vapour. For BAM-LL, BAM-P5L and BAM-LL in complex with Fab1, 3 μL of sample was applied to copper QUANTIFOIL R1.2/1.3 300 mesh, copper QUANTIFOIL R0.6/1 400 mesh and gold UltrAUfoil R1.2/1.3 300 mesh grids (Electron Microscopy Sciences), respectively, that had been glow discharged for 30 s at 60 mA in a GlowQube Plus (Electron Microscopy Sciences). Grids were blotted for 6 s with Whatman #1 filter paper at 4 °C and 80–100% relative humidity, before plunge freezing in liquid ethane using a Vitrobot Mark IV (ThermoFisher).

### CryoEM imaging

Data were collected on a 300 keV Titan Krios (ThermoFisher) EM in the Astbury Biostructure Laboratory in automated fashion using EPU software (ThermoFisher). Micrographs were recorded on an energy-filtered K2 detector (Gatan inc.) in counting mode, using a 100 μm objective aperture. For BAM-LL, 6456 micrographs were collected from a single grid over two sessions. For the Fab1-bound BAM-LL complex, 2780 micrographs were collected from a single grid. For BAM-P5L, two grids were imaged in separate sessions, resulting in 2150 total micrographs. For the BAM–Fab1 complex, a single grid was imaged over three sessions, resulting in 4197 total micrographs. Full data collection parameters for each sample are shown in Supplementary Table 4.

### Image processing

All processing was performed in RELION 3.0^66^ (BAM-LL, BAM–Fab1, Fab1-bound BAM-LL) or 3.1^67^ (BAM-P5L) unless otherwise stated. Dose-fractionated micrographs were motion-corrected and dose-weighted by MotionCor2^68^, before estimation of contrast transfer function parameters by Gctf^69^ using the motion corrected and dose-weighted micrographs, apart from the BAM–Fab1 complex where motion corrected, but non-dose-weighted, micrographs were used.

For BAM-LL, the two datasets were initially processed separately in a similar manner (Supplementary Fig. 8). For dataset 1, 299,458 particles were first picked using the general model in crYOLO 1.3.5^70^, and extracted in 300 pixel (321 Å) boxes with two-fold binning, before removal of false positives through two rounds of 2D classification. The resulting 234,598 particles were then used to generate an initial model by stochastic gradient descent^71^, which was used as the starting model for a 3D classification. Two high-resolution classes corresponding to different conformations of BAM-LL were obtained, one termed lateral-closed (86,615 particles) and one lateral-open (83,803 particles). Particles corresponding to each class were then re-extracted unbinned, and autorefined with a mask excluding bulk solvent. After masking and sharpening, resolutions of 5.0 Å (lateral-closed) and 5.9 Å (lateral-open) were obtained. Processing of dataset 2 proceeded similarly and resulted in comparable resolutions for both conformations. To achieve higher resolution, one round of CTF refinement followed by Bayesian polishing was then employed for each dataset, following which the particles corresponding to the same conformation were combined, resulting in 160,118 lateral-closed and 141,612 lateral-open particles. Finally these particle stacks were subject to separate non-uniform refinements in cryoSPARC v2.2.0^71,72^. Masking and sharpening of the resulting half-maps in RELION resulted in resolutions of 4.1 Å (lateral-closed) and 4.8 Å (lateral-open). B-factors of −107 and −127 Å^2^ were applied to the final lateral-closed and lateral-open reconstructions, respectively. Local resolution was estimated using RELION.

For the BAM–Fab1 complex (Supplementary Fig. 10), particles were autopicked in RELION 3^66^ using class averages from a previous reconstruction^8^ filtered to 30 Å as search templates. For each dataset, individual particles were extracted in 350 pixel (374.5 Å) boxes and culled with multiple rounds of 2D and 3D classification. At no point during this classification was a class consistent with the lateral-closed state identified. The final particle stacks from each dataset were then combined, resulting in 267,653 particles which could be refined to a resolution of 7.8 Å. It was then noticed that the density for BamB was weaker than the rest of the complex. A focused classification was therefore performed using a mask to only classify particles based on the region containing BamB. This resolved a lower resolution class mostly lacking BamB (135,778 particles, 10.4 Å), and a higher resolution class corresponding to the full complex (131,875 particles, 7.9 Å). Aside from occupancy of BamB, the two classes were conformationally identical, with both being entirely consistent with a lateral-open conformation. The particle stack for the higher resolution class was then refined, subject to a round of CTF refinement and particle polishing, before finally being further refined using the non-uniform refinement function in CryoSPARC v2.2.0^71,72^. The reconstruction was performed on independent subsets and final resolution of 5.2 Å determined by ‘gold standard’ FSC^73^ in RELION. A *B*-factor of −167 Å^2^ was applied to the final reconstruction.

For BAM-P5L (Supplementary Figs. 11 and 12), particles were picked in crYOLO 1.4.1 using the general model. For dataset 1: 41,316 particles were picked and extracted in a 280 pixel (300 Å) box, for dataset 2: 54,532 particles were picked and extracted into 352 pixel (300 Å) boxes. Both used twofold binning. The extracted particles were combined into a single dataset and the resulting 95,848 particles passed through 2D classification. The best 21,483 particles were used to construct an initial model by stochastic gradient descent^71^, which was used as a reference for 3D classification of the 43,280 good particles from 2D classification. The resulting 24,101 particles were autorefined, and re-extracted as unbinned particles and subject to 3D classification using the autorefined model as the reference. The resulting 19,044 particles were autorefined with a mask to a resolution of 10.3 Å. A *B*-factor of −671 Å^2^ was applied to the final reconstruction

For the Fab1-bound BAM-LL complex (Supplementary Fig. 13), particles were picked in crYOLO 1.4.1 using a model trained with 11 handpicked micrographs spanning the defoci range. The resulting 162,844 particles were extracted in 300 (321 Å) pixel boxes with twofold binning. One round of 2D classification was used to cull the particle set to 108,096 particles which was then subject to 3D classification, using an initial model generated by stochastic gradient descent^71^ from the best 32,645 particles in that stack as a template. From this 3D classification run, only one conformer was observed, corresponding to a lateral-open, BAM-LL bound to Fab1. The 71,675 particles in the highest resolution class were autorefined, re-extracted as unbinned particles and subjected to 3D classification using the autorefined model as the reference, further culling the particle stack. Autorefinement and sharpening of the resulting 61,777 good particles gave a resolution of 7.3 Å. Finally, one round of CTF refinement followed by Bayesian polishing was carried out, and the resulting particle stacks were subject to non-uniform refinement in cryoSPARC v2.2.0^71,72^. Masking and sharpening of the resulting half-maps in RELION resulted in a resolution of 7.1 Å. A *B*-factor of −274 Å^2^ was applied to the final reconstruction.

### CryoEM model building and refinement

For LL-BAM in the lateral-closed cryoEM map, an existing crystal structure of intact BAM in a lateral-closed conformation (PDB ID: 5D0O^6^) was first edited to both remove the two natural cysteines in BamA and to insert the LL disulfide bond. This starting model was fitted to the density as a rigid body in Chimera^74^, before performing several iterations of real-space refinement in PHENIX 1.14^75^ with secondary structure restraints followed by manual refinement in COOT^76^, until satisfactory geometry and fit between model and map was obtained as assessed using MolProbity^77^. The extracellular region of eL6 (BamA_675-702_), C-terminal globular domains of BamC (BamC_89-344_), and regions at the chain termini of BamABCDE were insufficiently resolved and were not modelled. The final model contains BamA_24-675,702-810_ BamB_31-391_, BamC_30-85_, BamD_27-244_, BamE_29-111_.

As the resolution of the other structures was insufficient for the above approach, molecular dynamics flexible fitting (MDFF)^40^ was used to flexibly fit these conformations. For BAM-LL lateral-open, cascade MDFF (cMDFF) simulations of the lateral-closed atomic model with BamA truncated after residue 809 were first used to derive an initial fit to the LL lateral-open cryoEM map. Here, a series of Gaussian blurred density maps were generated using the volutil function in VMD (halfwidths *σ* = 0, 1,…, 6 Å). The atomic model was then simulated in vacuum and subjected to an external potential derived from the most blurred density map, causing it to be flexibly fit into the density. 2 ps of minimisation followed by 100 ps of equilibration were run with a gscale of 1.0 defining the strength of the external potential derived from the density map. Consecutive 100 ps simulations were then run into maps of decreasing blurring, where the end coordinates from the previous simulation were used as input for the next, until reaching the unblurred map. At each step, isomerism, chirality and secondary structure restraints were applied. Several repeats were run, taking advantage of the stochastic nature of the simulation to generate different fits. Additionally, a second MDFF simulation was also run into the unblurred map using PDB-5LJO^8^ as a starting model, to derive better conformations for BamA_720-734_ and BamA_807,808_. These models were then manually combined to give best mainchain fit to the density, before minimising against the unblurred map for 40 ps. In the combined model, BamA_429-440_, corresponding to eL1 and the extracellular sides of β1 and β2, was fitting into micelle density rather than protein density due to the low resolution in this region. A final set of 500 ps MDFF simulations were therefore run with this combined model against the unblurred map, in which BamA_429-440_ was not subject to the external potential. The best fitting structure from these runs was then minimised for 40 ps against the unblurred map and real space refined in PHENIX 1.14^75^ with secondary structure restraints to generate the final atomic model.

For the Fab1-bound wild-type BAM complex, an initial model was created from the BAM complex PDB entry 5LJO^8^, with BamA_687-700_ from 5EKQ^5^, and the Fab1 crystal structure determined here (PDB 7BM5). The C-terminal globular domains of BamC were truncated, leaving only the lasso^78^ region (residues 25–83) resulting in a starting model containing BamA_24-806_, BamB_22-392_, BamC_25-83_, BamD_26-243_, and BamE_24-110_. The starting model was fitted into each EM density as a rigid body using UCSF Chimera^74^ and flexibly fit using cMDFF^40^. This was followed by real space refinement in PHENIX 1.14^75^ using secondary structure restraints to generate the final atomic model, with the Fab1 crystal structure used as a reference model to generate additional restraints.

For the Fab1-bound lid-locked BAM complex, the final lid-locked lateral-open structure and the Fab1 crystal structure were rigid body fitted into the EM density using UCSF Chimera and flexibly fit using a round of MDFF into the unblurred map. This was followed by real space refinement in PHENIX 1.14 with secondary structure restraints to generate the final atomic model, with the Fab1 crystal structure and the final lid-locked lateral-open structures used as reference models to generate additional restraints. During the simulation eL1 of BamA (BamA_429-440_) was not subject to the external potential to prevent overfitting to micelle density in this region. Model building statistics for all cryoEM conformers are shown in Supplementary Table 5.

### Crystallisation and structure determination of Fab1

Fab1 at 6.5 mg/mL was crystallised by the sitting drop vapour diffusion method in 96-well SWISSCI 3-drop plates at 20 °C. Drops consisted of 100 nL protein and 100 nL crystallisation solution were dispensed using a Mosquito robot (TTP Labtech). Crystals were grown in 0.16 M lithium chloride, 22% (w/v) PEG6000, 0.1 M MES pH 6.0 and were harvested after 21 days. Crystals were cryo-protected in the crystallisation solution supplemented with 20% (v/v) ethylene glycol before flash-cooling into liquid nitrogen. X-ray data were collected at Diamond Light Source on beamline I24 from a single cryo-cooled crystal (100 K) using an X-ray wavelength of 0.9795 Å and a Pilatus3 6M detector. Diffraction data were collected for a total of 180° up to a resolution of 2.5 Å with a 0.2° oscillation using an exposure time of 0.04 s at 100% transmission. Crystallographic data processed using CCP4 v.7.0.078. X-ray diffraction data were indexed and integrated by autoPROC and STARANISO^79^ and were scaled to 2.96 Å in Aimless^80^ using the I24 beamline autoprocessing pipeline. The crystals belonged to a monoclinic space group *P*12_1_1 with unit cell parameters *a* = 92.0 Å, *b* = 130.1 Å, *c* = 138.9 Å, *α* = 90.00°, *β* = 106.1°, *γ* = 90.00°. The structure was solved by molecular replacement using Phaser^81^ and the C_H_ domain of the anti-NFG Fab as the search model (PDB accession number 1ZAN^82^). Crystallographic refinement was performed using PHENIX-1.9^75,83^ and model building was carried out in *Coot*^76^. MolProbity^77^ was used for structure validation and quality assessment. The final model coordinates and structure factors are deposited in the PDB under the accession number 7BM5. Ramachandran statistics were 96.5% favoured, 3.4% allowed and 0.1% outliers. The average B-factor was 41 Å^2^.

### Reconstitution of BamA and different BAM complexes into DMPC proteoliposomes

DMPC (*di*C_14:0_PC), purchased as powder from Avanti Polar Lipids (Alabaster, AL), was dissolved in 80:20 (v/v) chloroform/methanol mixture at 25 mg/mL. Appropriate volumes were dried to thin films in clean Pyrex tubes at 42 °C under N_2_ gas, and were further dried by vacuum desiccation for >3 h. BAM WT, BAM-LL and BAM-P5L or a 2:1 (mol/mol) mixture of Fab1 and BAM in TBS pH 8.0, 0.05% (w/v) DDM were mixed with DMPC lipid solubilized in TBS pH 8.0, 0.05% (w/v) DDM at a lipid to protein ratio (LPR) of 1600:1 (mol/mol). For BamA, DMPC lipid was first solubilised in TBS pH 8.0, 0.1% (w/v) LDAO. Empty liposomes were prepared by mixing DDM-solubilised lipid with an equivalent volume of buffer. Dialysis was performed as described for the preparation of *E. coli* polar lipid proteoliposomes, except that a temperature of 30 °C was used (above the DMPC transition temperature). Following dialysis, the proteoliposomes were pelleted twice by ultracentrifugation at 100,000 × *g* for 30 min at 4 °C and resuspended in TBS pH 8.0. The proteoliposomes were then extruded with 21 passes through a 0.1 µm polycarbonate membrane using a mini-extruder (Avanti) pre-equilibrated at 30 °C. Following ultracentrifugation as before, proteoliposomes were resuspended in TBS pH 8.0, protein concentration was determined using a BCA assay (ThermoScientific) and successful reconstitution was confirmed using SDS–PAGE.

### Probing lipid disorder using laurdan

Laurdan (Cambridge Bioscience) dissolved in DMSO was added to a final concentration of 4.2 µM (final DMSO concentration of 0.15% (v/v)) to a 0.8 µM suspension of BAM-, BamA- or empty-DMPC proteoliposomes (LPR 1600:1 mol/mol). The proteoliposomes were incubated at 25 °C overnight to allow random partitioning of the laurdan probe into the membrane. Fluorescence emission was measured at 440 and 490 nm for a total time of 10 s following excitation of laurdan fluorescence at 340 nm in quartz cuvettes using a PTI QuantaMaster fluorimeter with a 1 nm bandwidth and 1 s integration time. Excitation and emission slit widths were set to 0.1 nm. Spectra were acquired at increasing temperature intervals from 6 to 40 °C, and to test reversibility, from 40 to 6 °C, allowing the sample to equilibrate at each temperature for 3 min. Generalised polarisation (GP)^46^ was calculated from the ratio of fluorescence intensity at 440 and 490 nm, averaged over the 10 s acquisition, using the formula GP = (*I*_440_ − *I*_490_)/(*I*_440_ + *I*_490_), and was plotted against temperature. Mid-points and gradients of the transitions were determined by calculating the first derivative of the curve.

### Reporting summary

Further information on research design is available in the Nature Research Reporting Summary linked to this article.

## Supplementary information

Supplementary Information

Reporting Summary

## Data Availability

The final density maps are deposited in the Electron Microscopy Data Bank (EMDB) under accession numbers EMD-12232 (BAM-LL lateral-closed), EMD-12262 (BAM-LL lateral-open), EMD-12272 (BAM–Fab1 complex), EMD-12263 (BAM-P5L) and EMD-12271 (BAM-LL:Fab1 complex). Final model coordinates have been deposited in the Protein Data Bank (PDB) under accession numbers 7BNQ (BAM-LL lateral-closed), 7NBX (BAM-LL lateral-open), 7ND0 (BAM–Fab1 complex) and 7NCS (BAM-LL:Fab1 complex). The crystal structure of Fab1 has been deposited in the PDB under accession number 7BM5. Source data are provided with this paper.

## Source data

Source Data
