# Supplementary material for: The role of membrane destabilisation and protein dynamics in BAM catalysed OMP folding
Source: Nat Commun. 2021 Jul 7;12:4174. doi: 10.1038/s41467-021-24432-x (PMC8263589; doi:10.1038/s41467-021-24432-x)
Supplement: Supplementary file 1 — Supplementary Information [file 41467_2021_24432_MOESM1_ESM.pdf]

Supplementary Information

The role of membrane destabilisation and protein dynamics in BAM catalysed OMP folding

Paul White[1○‡], Samuel F. Haysom[1‡], Matthew G. Iadanza[1•‡], Anna J. Higgins[1], Jonathan M. Machin[1], James M. Whitehouse[1], Jim E. Horne[1#], Bob Schiffrin[1], Charlotte Carpenter-Platt[1], Antonio N. Calabrese[1], Kelly M. Storek[2], Steven T. Rutherford[2], David J. Brockwell[1], Neil A. Ranson[1*] and Sheena E. Radford[1*]

[1] Astbury Centre for Structural Molecular Biology, School of Molecular and Cellular Biology, Faculty of Biological Sciences, University of Leeds, Leeds, LS2 9JT, UK

[2] Department of Infectious Diseases, Genentech Inc., South San Francisco, CA 94080

[‡] Contributed equally

○ Current affiliation: GlaxoSmithKline R&D, Gunnelswood Road, Stevenage, SG1 2NY, UK

• Current affiliation: Scientific Computing Department, Science and Technology Facilities Council, Research Complex at Harwell, Didcot, OX11 0FA, UK

[#] Current affiliation: Department of Biochemistry, University of Oxford, Oxford, OX1 3QU, UK

*Correspondence: n.a.ranson@leeds.ac.uk; s.e.radford@leeds.ac.uk

Supplementary Table 1 | Initial folding rates for tOmpA and OmpX for each BAM variant. OMP substrates were folded into *E. coli* polar lipid proteoliposomes in TBS pH 8.0 at 25 °C in the presence of the various BAM variants. Each experiment was repeated at least once, as stated to confirm reproducibility (note that more replicates were included for WT BAM as this construct was included as a control when each variant was assayed. Initial rates were calculated independently for each replicate with the range of values (maximum – minimum) shown to highlight reproducibility. The initial rates presented show the average of (n) repeat experiments. Initial rates as a percentage of the WT BAM initial rate are also shown for each variant.

	tOmpA				OmpX			
**Variant**	Initial rate (s^{-1})	Range (s^{-1})	Number of replicates (n)	Rel. to WT BAM (%)	Initial rate (s^{-1})	Range (s^{-1})	Number of replicates (n)	Rel. to WT BAM (%)
WT BAM	7.12×10^{-4}	5.81×10^{-5}	4	100	2.20×10^{-4}	1.24×10^{-4}	7	100
BAM-Fab1	1.16×10^{-4}	1.79×10^{-5}	2	16	1.87×10^{-5}	2.89×10^{-6}	2	8
BAM-P5L	1.45×10^{-4}	6.69×10^{-5}	2	20	6.34×10^{-5}	5.14×10^{-5}	2	29
BAM-P5L + DTT	1.30×10^{-3}	3.40×10^{-5}	2	183	5.74×10^{-4}	1.19×10^{-4}	2	261
BAM-LL	1.32×10^{-4}	5.20×10^{-6}	2	19	2.84×10^{-5}	1.83×10^{-5}	3	13
BAM-LL + DTT	1.35×10^{-3}	6.70×10^{-5}	2	190	3.46×10^{-4}	1.13×10^{-4}	3	157
BamA	2.60×10^{-5}	1.00×10^{-5}	2	4	6.51×10^{-6}	3.23×10^{-6}	2	3
Empty	0.00	0.00	2	0	0.00	0.00	2	0
BAM-P5L + Fab1	2.50×10^{-5}	1.09×10^{-5}	2	4	1.23×10^{-5}	2.60×10^{-6}	2	6
BAM-LL + Fab1	9.73×10^{-6}	2.00×10^{-9}	2	1	2.80×10^{-6}	1.50×10^{-6}	2	1
WT BAM + DTT	6.99×10^{-4}	4.48×10^{-4}	2	98	ND	ND	ND	ND
Cys-free BAM	8.11×10^{-4}	3.82×10^{-4}	5	114	ND	ND	ND	ND
Cys-free BAM + DTT	9.68×10^{-4}	3.04×10^{-4}	4	136	ND	ND	ND	ND

Supplementary Table 2 | Folding yields for tOmpA and OmpX after 24 hours for inhibited BAM variants, BamA and empty liposomes. Folding yield data are reported as the mean of two biological replicates, except for BAM-Fab1 folding tOmpA which was performed once only. The range of values covered by the replicates is also reported.

**Variant**	**tOmpA**		**OmpX**	
	Folding yield after 24 hours (%)	Range (%)	Folding yield after 24 hours (%)	Range (%)
**BAM-P5L**	100	0	92	8
**BAM-LL**	96	5	88	0
**BAM-Fab1**	84	-	50	9
**BAM-P5L + Fab1**	67	7	52	19
**BAM-LL + Fab1**	52	11	11	8
**BamA**	50	3	14	5
**Empty**	0	0	0	0

Supplementary Table 3 | X-ray diffraction data processing and model refinement statistics for Fab1.

	7BM5
**Data collection**	
Space group	$P\ 1\ 2_1\ 1$
Cell dimensions	
a, *b*, *c* (Å)	92.01, 130.14, 138.92
α, β, γ (°)	90.00, 106.06, 90.00
Resolution (Å)	93.19-2.96 (3.43-2.96) *
R_{sym} or R_{merge}	0.422 (1.058)
$I / \sigma I$	3.6 (1.6)
Completeness (%)	92.1 (68.8)
Redundancy	6.8
**Refinement**	
Resolution (Å)	88.42-2.96 (3.05-2.96)
No. reflections	30473
R_{work} / R_{free}	25.7/29.0
No. atoms	18443
Protein	18443
Ligand/ion	0
Water	0
B-factors	(Ask for input)
Protein	41.56
Ligand/ion	
Water	
R.m.s. deviations	
Bond lengths (Å)	0.0055
Bond angles (°)	0.992

*Values in parentheses are for highest-resolution shell.

Supplementary Table 4 | CryoEM data processing and model building statistics

**Sample**	**Lid-lock BAM**		**POTRA-lock BAM**		**wild-type BAM-Fab1 complex**			**lid-lock BAM-Fab1 complex**
Buffer	TBS[a], 0.05 % DDM		TBS[a], 0.05 % DDM		TBS[b], 0.05 % DDM			TBS[b], 0.03 % DDM
Dataset	1	2	1	2	1	2	3	1
Microscope	FEI Titan Krios	FEI Titan Krios	FEI Titan Krios	FEI Titan Krios	FEI Titan Krios	FEI Titan Krios	FEI Titan Krios	FEI Titan Krios
Camera	Gatan K2	Gatan K2	Gatan K2	Gatan K2	Gatan K2	Gatan K2	Gatan K2	Gatan K2
Magnification (x)	130,000	130,000	130,000	165,000	130,000	130,000	130,000	130,000
Voltage (kV)	300	300	300	300	300	300	300	300
Micrographs	2542	3914	686	1464	663	43	3491	2780
Exposure time frame/total (s)	0.25/8	0.25/8	0.22/7	0.13/6	0.2/10	0.2/10	0.2/10	0.22/7
Number of frames per image	32	32	32	48	50	50	50	32
Electron exposure per frame/total (e^{-}/Å2)	1.55/49.7	1.55/49.7	1.56/50.0	1.2/57.6	1.49/74.9	1.19/59.8	1.22/60.9	1.53/49.1
Defocus range (μm)	-1.5 to -3	-1 to -2.5	-1 to -2.5	-1 to -2.5	-1.75 to -3.25	-1.75 to -3.25	-1.75 to -3.25	-1 to -2.5
Pixel size (Å)	1.07	1.07	1.07	0.85	1.07	1.07	1.07	1.07

[a] TBS: 50 mM Tris-HCl pH 8.0, 150 mM NaCl

[b] TBS: 20 mM Tris-HCl pH 8.0, 150 mM NaCl

Supplementary Table 5 | CryoEM model building statistics

**Sample**	**lid-locked BAM**		**wild-type BAM-Fab1 complex**	**lid-locked BAM-Fab1 complex**
Conformation	lateral-closed	lateral-open (contorted)	lateral-open	lateral-open (contorted)
Accession codes	(EMD-12232, PDB-7BNQ)	(EMD-12262, PDB-7NBX)	(EMD-12272, PDB-7ND0)	(EMD-12271, PDB-7NCS)
Box size (pixels)	300		350	300
Symmetry imposed	C1		C1	C1
Initial particle images (no.)	793,444		703,997	162,844
Final particle images (no.)	160,118	141,612	131,853	61,777
Map resolution (Å) 0.143 FSC threshold	4.1	4.8	5.2	7.1
Map resolution range[1] (Å)	3.9 - 7.0	4.5 - 8.5	4.9 - 11.2	5.9 - 16.0
Initial model used (PDB code)	5D0O	lid-locked BAM lateral-closed conformation (7BNQ), 5LJO	5LJO, 5EKQ, Fab1 (7BM5)	lid-locked BAM lateral-closed conformation (7BNQ), Fab1 (7BM5)
Model resolution (Å)	4.1	4.8	5.3	7.2
FSC threshold	0.5	0.5	0.5	0.5
Map sharpening *B* factor (Å^2)	-107	-127	-167	-274
Model composition				
Nonhydrogen atoms	11617	11514	15243	14843
Protein residues	1488	1475	1960	1911
Ligands	0	0	0	0
B factors (Å^2)				
Protein	103.1	210.52	241.90	183.27
R.m.s. deviations				
Bond lengths (Å)	0.005	0.006	0.006	0.009
Bond angles (°)	0.79	0.923	1.050	1.39
Validation				
MolProbity score	1.63	2.21	2.17	2.29
Clashscore	4.99	12.41	11.47	15.59
Poor rotamers (%)	0.32	0	0.24	0.56
Ramachandran plot				
Favored (%)	94.65	87.61	88.34	88.17
Allowed (%)	5.35	12.25	11.25	11.04
Disallowed (%)	0	0.14	0.41	0.79

[1]The total range of local resolution values (calculated in RELION) within the mask used to calculate the global FSC.

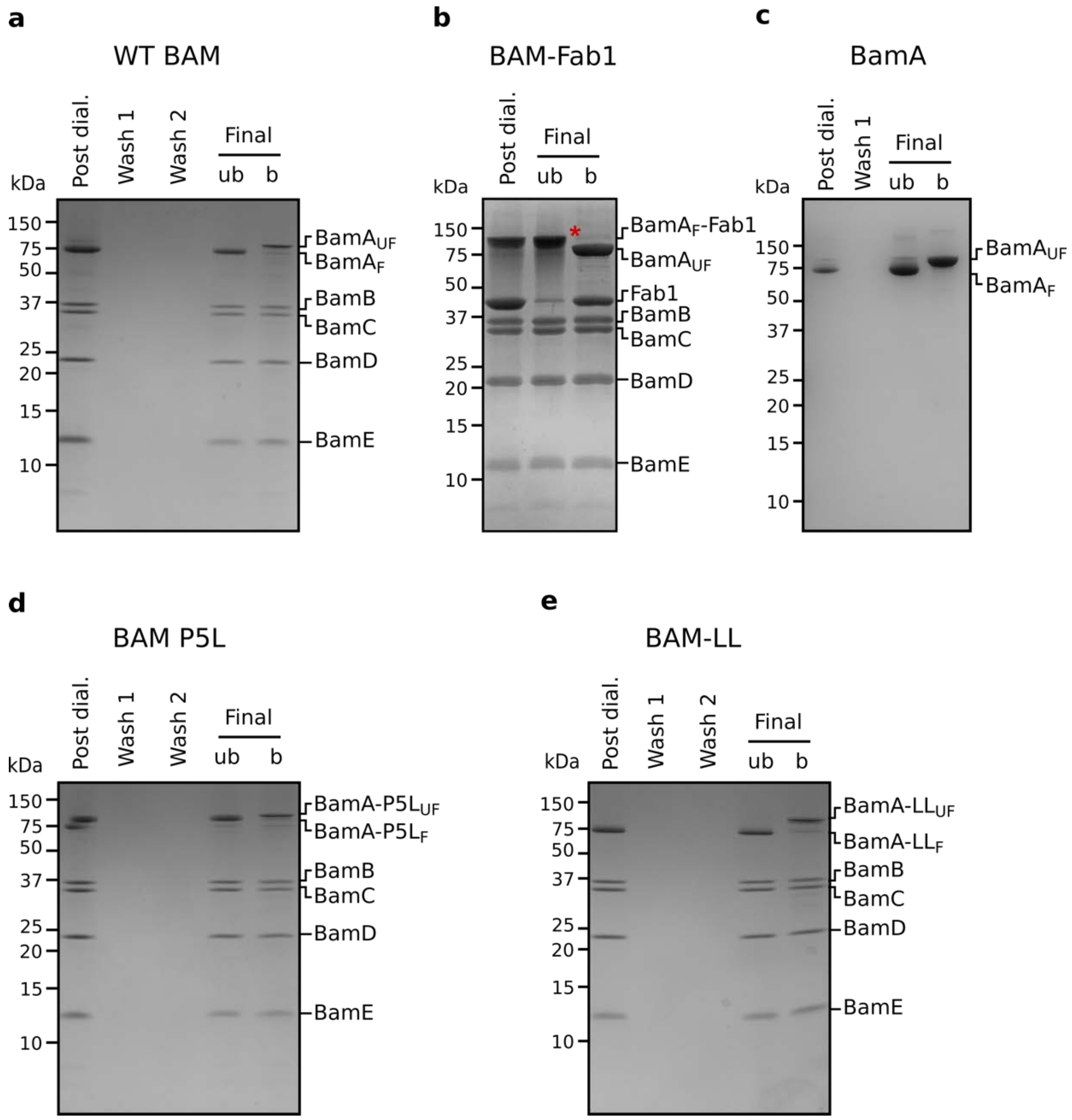

Supplementary Figure 1 | SDS-PAGE analysis of BAM/BamA in *E. coli* polar lipid proteoliposomes. Quality of *E. coli* polar lipid proteoliposomes prepared by dialysis (see Methods) containing **(a)** WT BAM, **(b)** BAM-Fab1, **(c)** BamA, **(d)** BAM-P5L or **(e)** BAM-LL were assessed by SDS-PAGE. All five BAM proteins (and Fab1 for BAM-Fab1 complex proteoliposomes) were present and a mobility shift between boiled and un-boiled samples (*b and ub, respectively*) for BamA was observed, distinguishing folded BamA (BamA$_F$) from unfolded BamA (BamA$_{UF}$). In (**b**), an SDS-resistant complex between BamA and Fab1 can be observed, running as a single band on the gel (*). Samples of proteoliposomes immediately following dialysis (*post dial.*) along with up to two wash samples (*see Methods*) were loaded to confirm successful reconstitution. 15 µL samples were mixed with 5 µL SDS-PAGE sample buffer and 15 µL was loaded on the gel. Boiled samples were heated for 10 min at > 95 °C prior to loading. This experiment was performed once for every proteoliposome variant produced for this study. Source data for all figure parts are provided as a source data file.

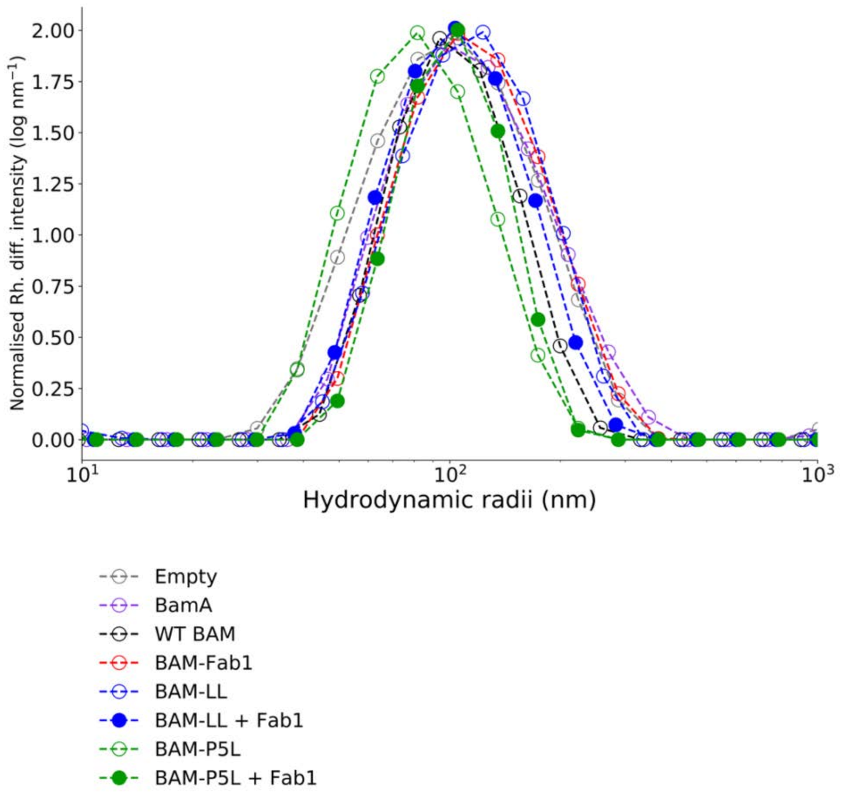

Liposome/ Proteoliposome	Mean r_H (nm)	Standard deviation of r_H (nm)
**Empty**	109	10
**BamA**	104	15
**WT BAM**	111	7
**BAM-Fab1**	123	4
**BAM-LL**	118	5
**BAM-LL + Fab1**	105	7
**BAM-P5L**	90	3
**BAM-P5L + Fab1**	112	10

Supplementary Figure 2 | Dynamic light scattering of all proteoliposome variants used in folding assays. Representative data for *E. coli* polar lipid empty liposomes (*grey, open circles*) and proteoliposomes containing BamA (*purple, open circles*), WT BAM (*black, open circles*), BAM-Fab1 (*red, open circles*), BAM-LL (*blue, open circles*), BAM-LL + Fab1 (*blue, filled circles*), BAM-P5L (*green, open circles*) and BAM-P5L + Fab1 (*green, filled circles*). The intensities have been normalised to allow peak-shape comparison. The average mean hydrodynamic radii (r_H) and the standard deviation from three replicate measurements are reported in the *inset table*. All liposome/proteoliposomes have an average r_H ranging from 90-123 nm. This experiment was performed once for every proteoliposome variant produced for this study. Source data are provided as a source data file.

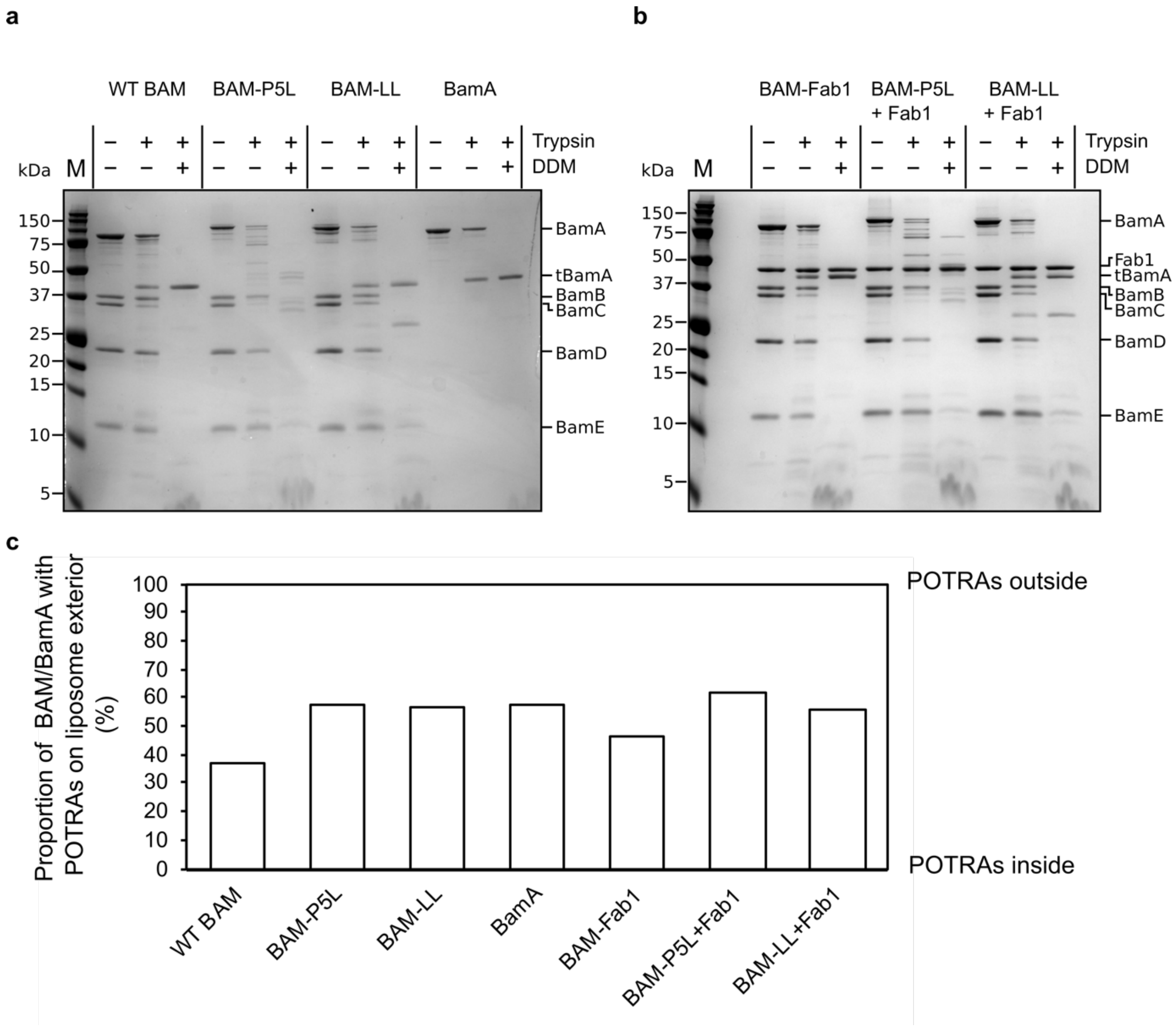

Supplementary Figure 3 | Orientation of BAM/BamA is similar in the proteoliposome variants. BAM complex variants and BamA in the *E. coli* polar lipid proteoliposomes used in folding assays were digested with trypsin to determine orientation. POTRA domains and BAM lipoproteins (BamBCDE) on the exterior side of the liposome bilayer represents the substrate accessible orientation (equivalent to BAM's periplasmic face) and are sensitive to trypsin proteolysis, whilst the BamA extracellular loops are protected inside the liposome. Proteoliposomes were incubated with trypsin at a protein:trypsin ratio of 50:1 (*w/w*), at 37 °C for 16 h in TBS pH 8.0 *(see Methods).* A non-digested control was included, as well as a proteolysis experiment in the presence of 1% (*w/v*) DDM to permeabilise the liposomes. Digest reactions were halted by boiling in SDS-PAGE loading buffer and analysed by SDS-PAGE. Each experiment was performed once. **(a)** SDS-PAGE analysis of proteolysis experiments on WT BAM, BAM-P5L, BAM-LL and BamA. **(b)** SDS-PAGE analysis of proteolysis experiments on BAM-Fab1, BAM-P5L + Fab1 and BAM-LL + Fab1. **(c)** SDS-PAGE bands from a) and b) for remaining intact BAM/BamA after incubation with trypsin were quantified by densitometry to determine the proportion of lipoproteins and POTRAs on the exterior side (*see Methods*). Source data for all figure parts are provided as a source data file.

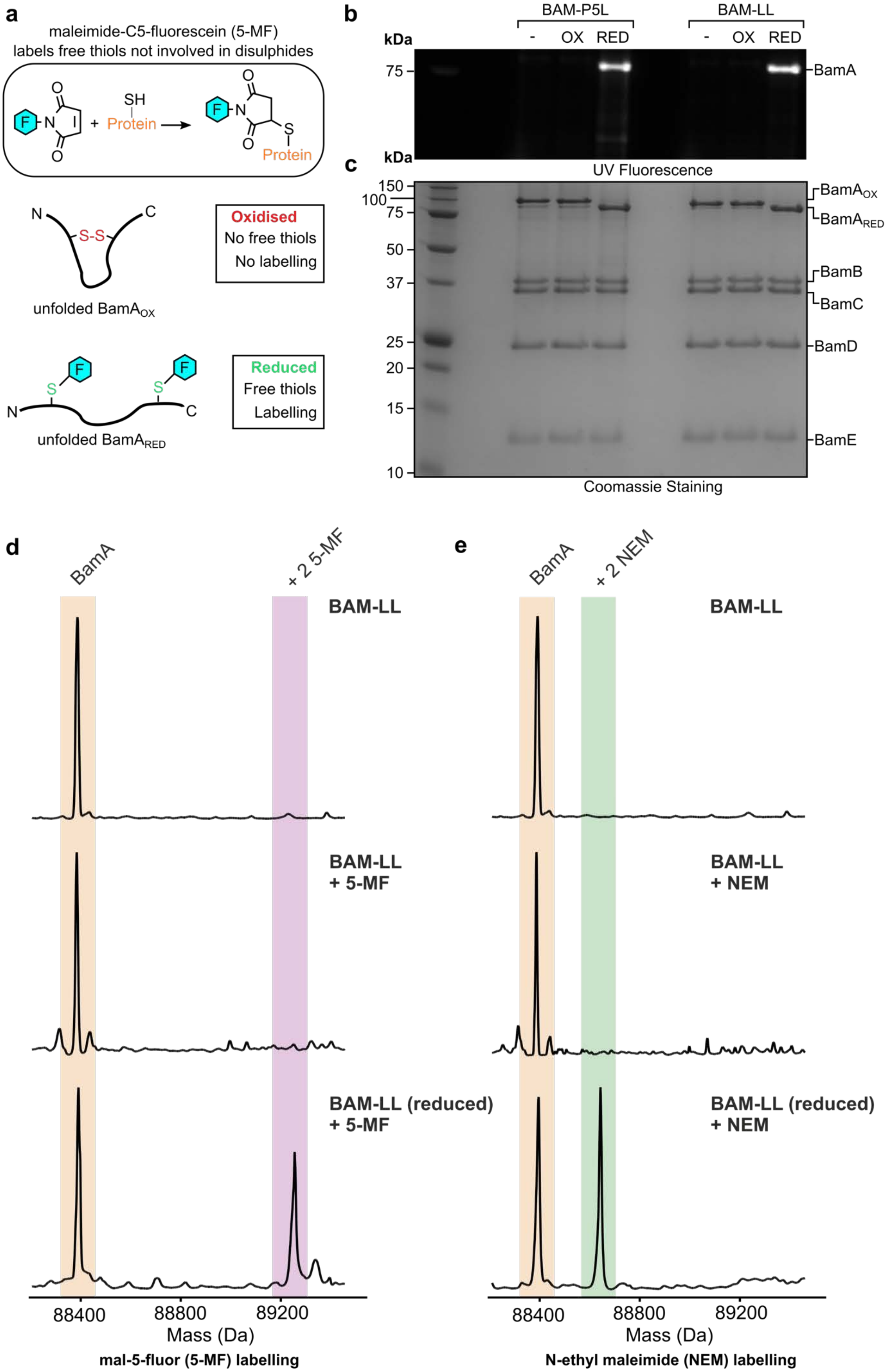
a
maleimide-C5-fluorescein (5-MF)
labels free thiols not involved in disulphides
SH
Protein
Protein
Oxidised
No free thiols
No labelling
unfolded BamA$_{OX}$
Reduced
Free thiols
Labelling
unfolded BamA$_{RED}$
b
BAM-P5L
BAM-LL
- OX RED
- OX RED
kDa
75
BamA
UV Fluorescence
c
kDa
150
100
75
50
37
25
20
15
10
BamA$_{OX}$
BamA$_{RED}$
BamB
BamC
BamD
BamE
Coomassie Staining
d
BamA
+ 2 5-MF
BAM-LL
BAM-LL
+ 5-MF
BAM-LL (reduced)
+ 5-MF
88400
88800
89200
Mass (Da)
mal-5-fluor (5-MF) labelling
e
BamA
+ 2 NEM
BAM-LL
BAM-LL
+ NEM
BAM-LL (reduced)
+ NEM
88400
88800
89200
Mass (Da)
N-ethyl maleimide (NEM) labelling

Supplementary Figure 4 | Verifying formation of the disulphide bond in BAM-LL and BAM-P5L by maleimide labelling of denatured BAM. (a) The maleimide moiety only reacts with reduced cysteine thiol groups (-SH) and not those involved in a disulphide bond (-S-S-). **(b)** To show the extent of labelling of BamA in each BAM complex, urea-denatured, labelled samples were separated by SDS-PAGE and visualised by UV fluorescence. Samples were labelled with maleimide-5-fluorescein (5-MF) without treatment (-), or after incubation under oxidising (100 □M diamide, OX) or reducing (1 mM TCEP, RED) conditions (controls for disulphide formation and reduction, respectively). Quantitation of the bands confirmed that at least 97% of molecules have an intact disulphide band. **(c)** Following fluorescence imaging, the gel was stained with Coomassie Blue to show the total protein content. The mobility of BamA in untreated samples is identical to the protein treated with diamide (oxidised) and distinct from that of reduced samples, confirming complete disulphide bond formation in these samples. Samples were not boiled prior to loading. Each experiment was repeated at least once. Molecular weight markers are shown on the left hand side of each gel (kDa). Consistent with results from the gels, mass spectrometric analysis of denatured BAM-LL after incubation with either **(d)** 5-MF or **(e)** N-ethyl maleimide (NEM) showed no labelling of BamA. The deconvoluted intact ESI mass spectra of BamA obtained after incubation in the absence or presence of the labelling reagent were identical (top and middle panels in **d** and **e**). Only after reduction with 1 mM TCEP prior to labelling (bottom panels in **d** and **e**), were ESI mass spectra with higher mass peaks corresponding to addition of two labels seen (consistent with labelling of both reduced cysteine residues). Guanidine HCl was substituted for urea in these experiments to avoid protein carbamylation which would have complicated the analysis. This experiment was performed once. Source data are provided as a source data file.

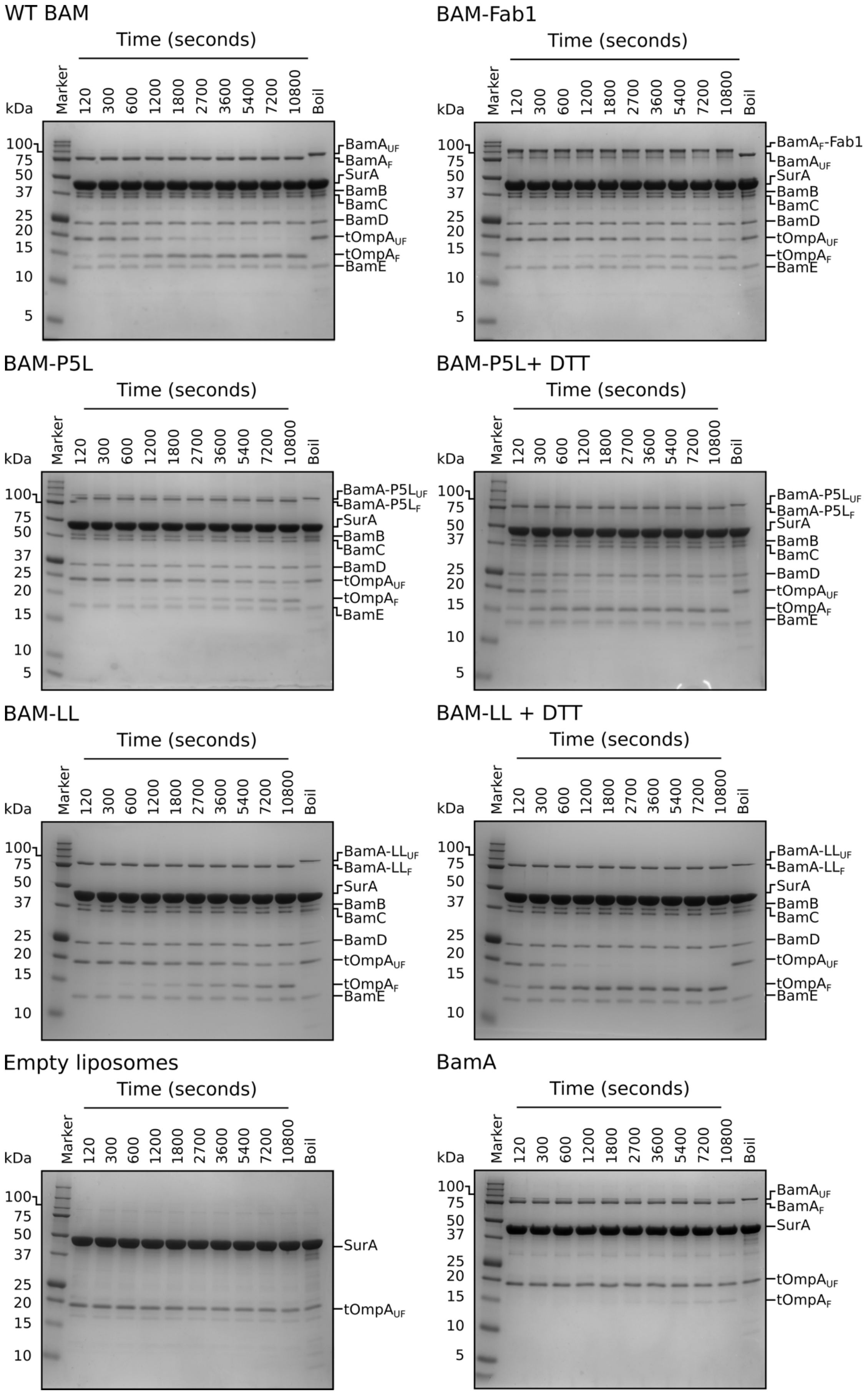
WT BAM
Time (seconds)
Marker
120
300
600
1200
1800
2700
3600
5400
7200
10800
Boil
kDa
100
75
50
37
25
20
15
10
5
BamA$_{UF}$
BamA$_{F}$
SurA
BamB
BamC
BamD
tOmpA$_{UF}$
tOmpA$_{F}$
BamE
BAM-Fab1
BamA$_{F}$-Fab1
BamA$_{UF}$
BAM-P5L
BamA-P5L$_{UF}$
BamA-P5L$_{F}$
BAM-P5L+ DTT
BAM-LL
BamA-LL$_{UF}$
BamA-LL$_{F}$
BAM-LL + DTT
Empty liposomes
BamA

Supplementary Figure 5 | SDS-PAGE band-shift folding assays for tOmpA. Folding of tOmpA by BAM variants was assayed by SDS-PAGE band-shift assays, as described previously[1], where folded/unfolded species have different electrophoretic mobilities. Folding reactions contained 2 µM tOmpA, 10 µM SurA and 1 µM BAM-containing *E. coli* polar lipid proteoliposomes in TBS pH 8.0, 0.8 M urea, along with 25 mM DTT for experiments containing reducing agent. Reactions were incubated at 25 °C and samples for SDS-PAGE analysis were taken at time intervals that were subsequently run on 15% (*w/v*) Tris-tricine SDS-PAGE gels at room temperature. A fully unfolded control (*Boil*) was included by heating the sample for 10 min at > 95 °C prior to loading. Band intensities for folded and unfolded tOmpA ($tOmpA_F$ and $tOmpA_{UF}$, respectively) were quantified to determine the fraction folded at a given time point. Each experiment was repeated at least twice to confirm reproducibility. The number of replicates is shown in Supplementary Table 1. Source data for all figure parts are provided as a source data file.

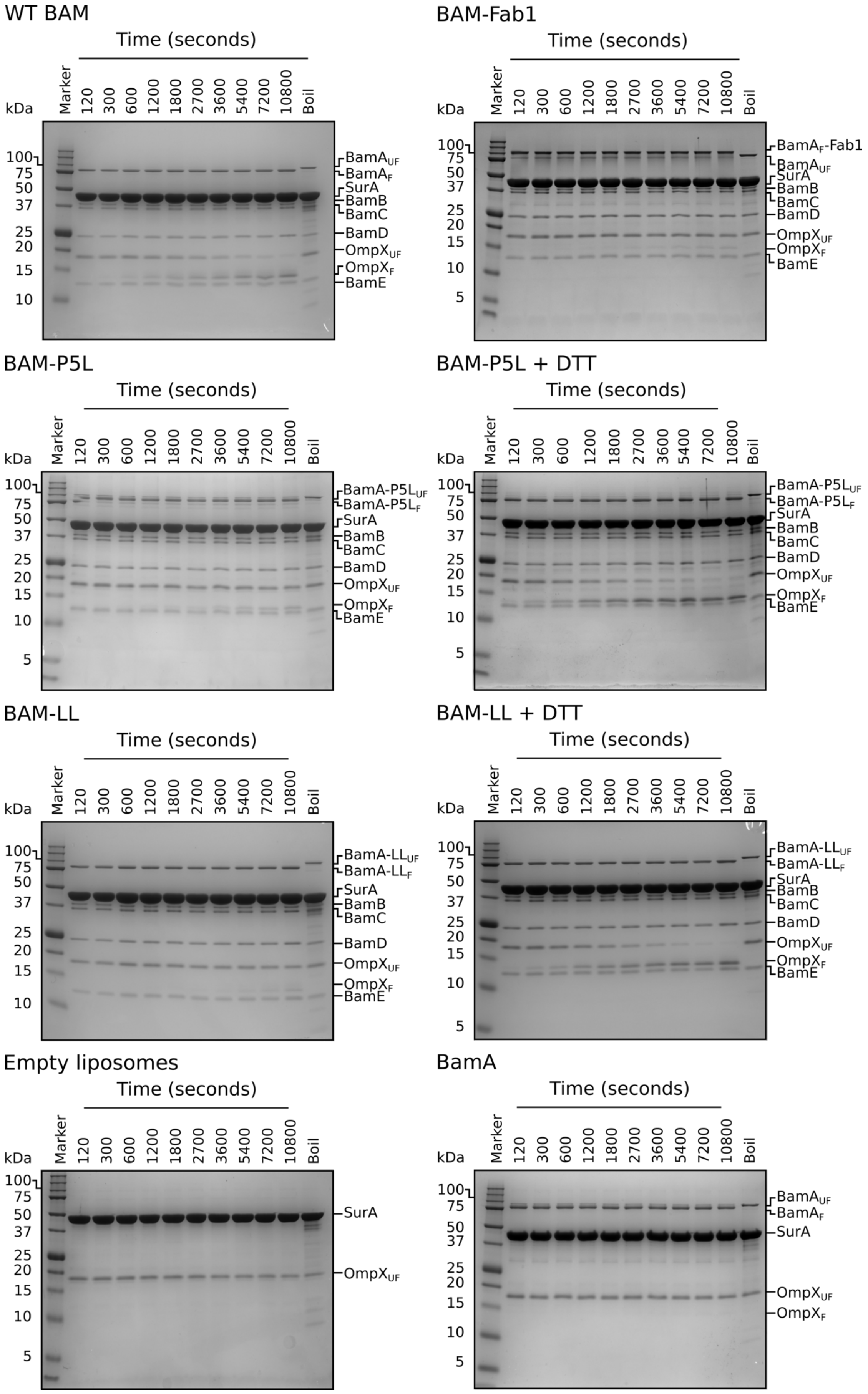
WT BAM
BAM-Fab1
BAM-P5L
BAM-P5L + DTT
BAM-LL
BAM-LL + DTT
Empty liposomes
BamA
Time (seconds)
kDa
Marker
120
300
600
1200
1800
2700
3600
5400
7200
10800
Boil
100
75
50
37
25
20
15
10
5
BamA$_{UF}$
BamA$_{F}$
BamA$_{F}$-Fab1
BamA-P5L$_{UF}$
BamA-P5L$_{F}$
BamA-LL$_{UF}$
BamA-LL$_{F}$
SurA
BamB
BamC
BamD
OmpX$_{UF}$
OmpX$_{F}$
BamE

Supplementary Figure 6 | SDS-PAGE band-shift folding assays for OmpX. Assays were conducted as described for tOmpA (see Supplementary Fig. 5) except that reactions contained 2 µM OmpX instead of tOmpA as the folding substrate. Band intensities for folded and unfolded OmpX ($OmpX_F$ and $OmpX_{UF}$, respectively) were quantified to determine the fraction folded at a given time point. Each experiment was repeated at least twice to confirm reproducibility. The number of replicates is shown in Supplementary Table 1. Source data for all figure parts are provided as a source data file.

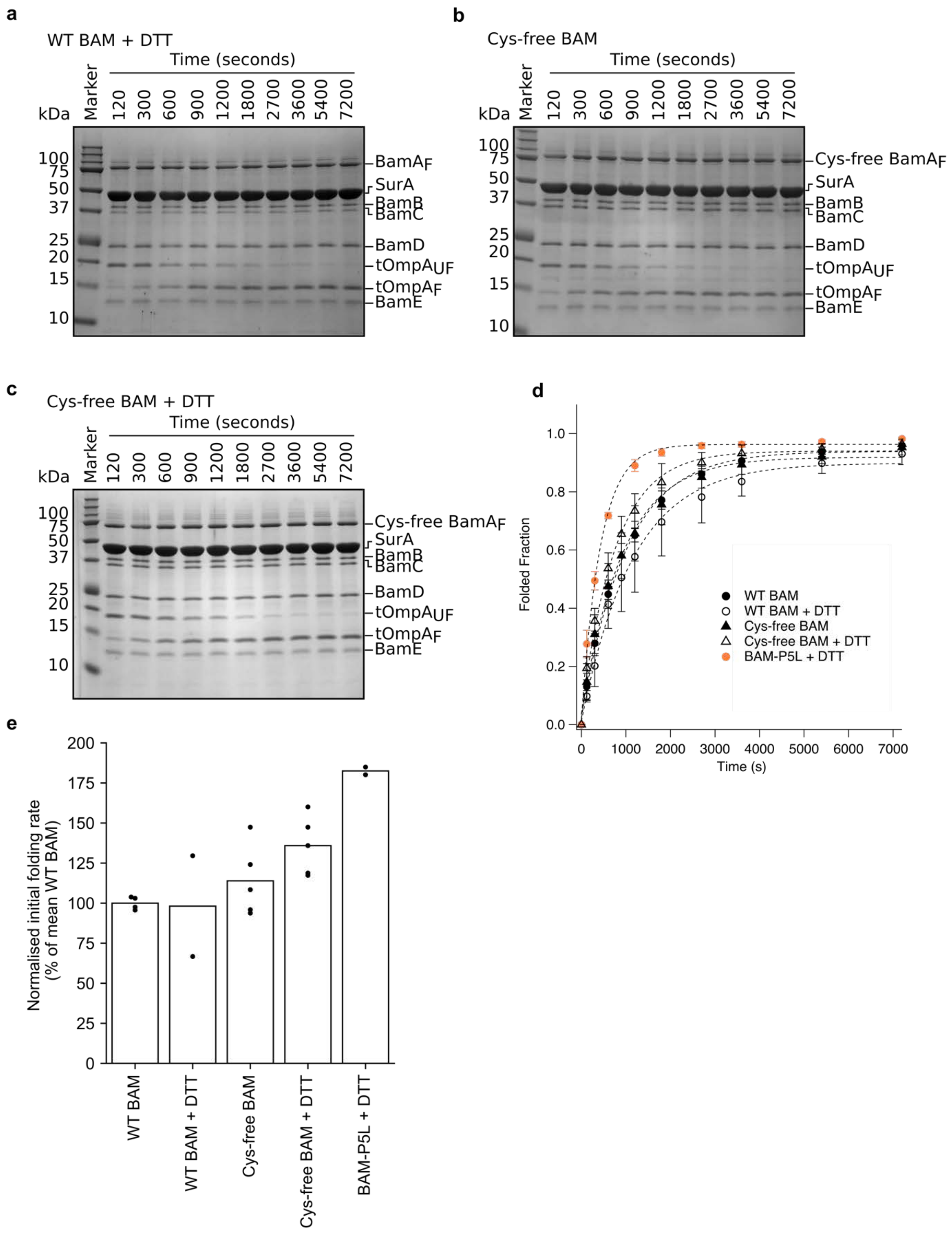

Supplementary Figure 7 | Mutation of the two natural cysteines in BamA has little effect on tOmpA folding rate. SDS-PAGE band-shift folding assays were carried out for **(a)** WT-BAM + 25 mM DTT, **(b)** Cys-free BAM (C690S/C700S) and **(c)** Cys-free BAM (C690S/C700S) + 25 mM DTT using tOmpA as a substrate and the same protocol as for other BAM variants (*see Supplementary Figure 5, and Methods*). Band intensities for folded and unfolded tOmpA (tOmpA$_F$ and tOmpA$_{UF}$, respectively)

were quantified to determine the fraction folded at a given time point. **(d)** Average fraction folded determined from the gels shown in **(a-c)** plotted against time. Traces for WT BAM and BAM-P5L are shown for comparison (using data shown in *Supplementary Fig. 5*). The data show that the removal of the two natural cysteines, or addition of DTT, have little effect on folding rate for tOmpA relative to WT BAM, while reduced BAM-P5L displays more rapid tOmpA folding relative to WT. Data markers represent the average folded fractions calculated from at least two repeats (The number of replicates is shown in Supplementary Table 1) and dashed lines are single exponential fits of the data. Error bars represent the range of values covered by the replicates. **(e)** The initial rates of folding were determined by applying a linear fit to the first 5% of folding data shown in **(d)**, and were normalised as a percentage of the mean initial rate obtained for WT BAM. Bars represent the mean value for each condition, with values for each replicate shown as grey points. Source data for all figure parts are provided as a source data file.

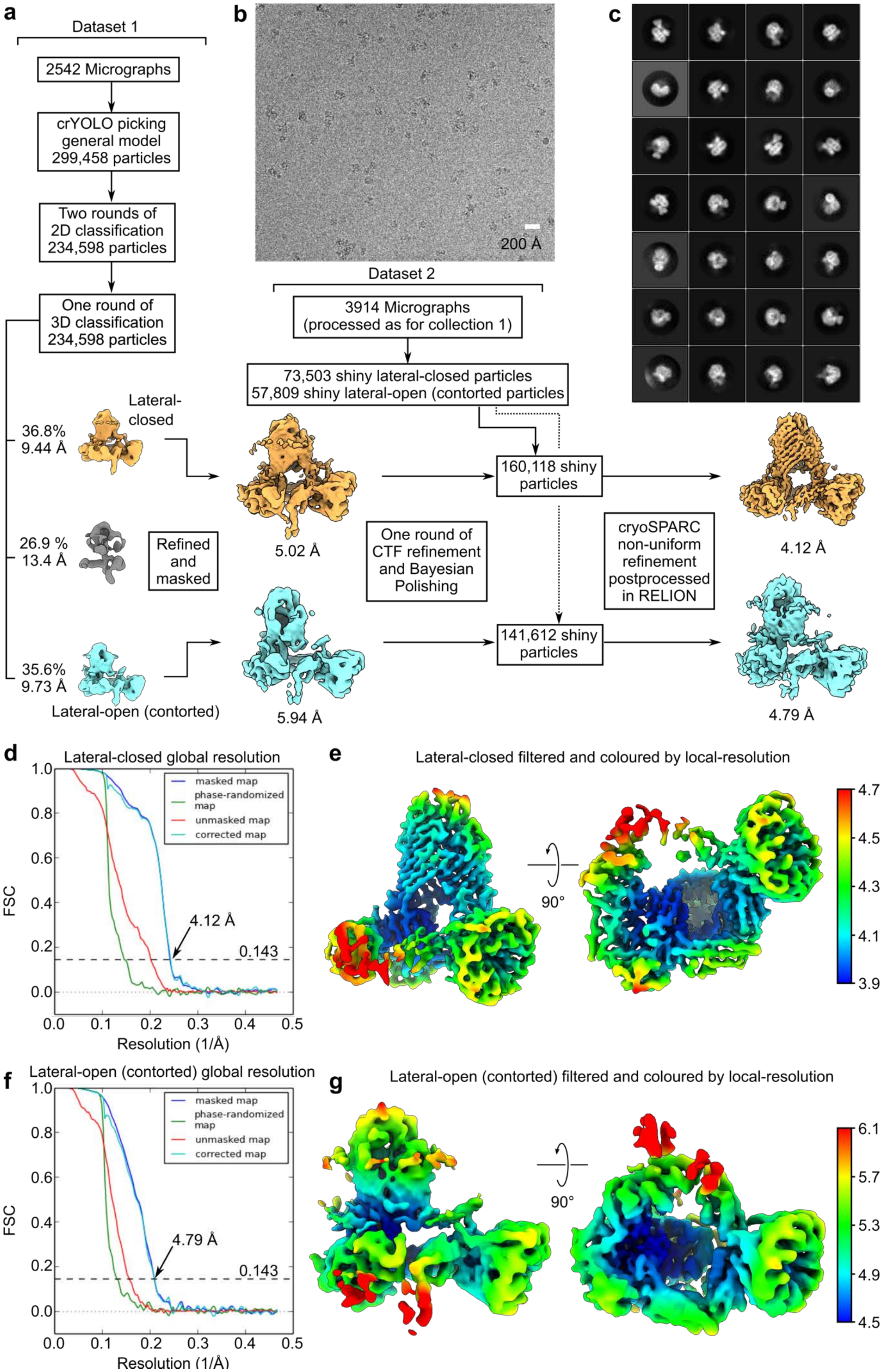
a
Dataset 1
2542 Micrographs
crYOLO picking
general model
299,458 particles
Two rounds of
2D classification
234,598 particles
One round of
3D classification
234,598 particles
Lateral-
closed
36.8%
9.44 Å
26.9 %
13.4 Å
Refined
and
masked
35.6%
9.73 Å
Lateral-open (contorted)
b
200 Å
Dataset 2
3914 Micrographs
(processed as for collection 1)
73,503 shiny lateral-closed particles
57,809 shiny lateral-open (contorted particles
5.02 Å
5.94 Å
One round of
CTF refinement
and Bayesian
Polishing
160,118 shiny
particles
141,612 shiny
particles
cryoSPARC
non-uniform
refinement
postprocessed
in RELION
4.12 Å
4.79 Å
c
d
Lateral-closed global resolution
masked map
phase-randomized
map
unmasked map
corrected map
FSC
4.12 Å
0.143
Resolution (1/Å)
e
Lateral-closed filtered and coloured by local-resolution
90°
4.7
4.5
4.3
4.1
3.9
f
Lateral-open (contorted) global resolution
masked map
phase-randomized
map
unmasked map
corrected map
FSC
4.79 Å
0.143
Resolution (1/Å)
g
Lateral-open (contorted) filtered and coloured by local-resolution
90°
6.1
5.7
5.3
4.9
4.5

Supplementary Figure 8 | Solving the structures of BAM-LL. (a) Image processing workflow for BAM-LL in DDM detergent. Micrographs from dataset 2 were processed similarly to dataset 1, with polished and CTF corrected (shiny) particles from both datasets being combined and subject to non-uniform refinement in cryoSPARC v2.2.0 to give the final reconstructions. The structure determination process was performed once. **(b)** Representative micrograph (321 Å box) and **(c)** Representative 2D classes from collection 2. **(d)** FSC plot, calculated in RELION, used to estimate global resolution for the final lateral-closed reconstruction. **(e)** Final lateral-closed reconstruction, filtered and coloured by local-resolution as calculated in RELION. Map contour is 11.5 σ. **(f)** FSC plot, calculated in RELION, used to estimate global resolution for the final lateral open (contorted) reconstruction. **(g)** Final lateral open (contorted) reconstruction, filtered and coloured by local-resolution as calculated in RELION. Map contour is 8 σ.

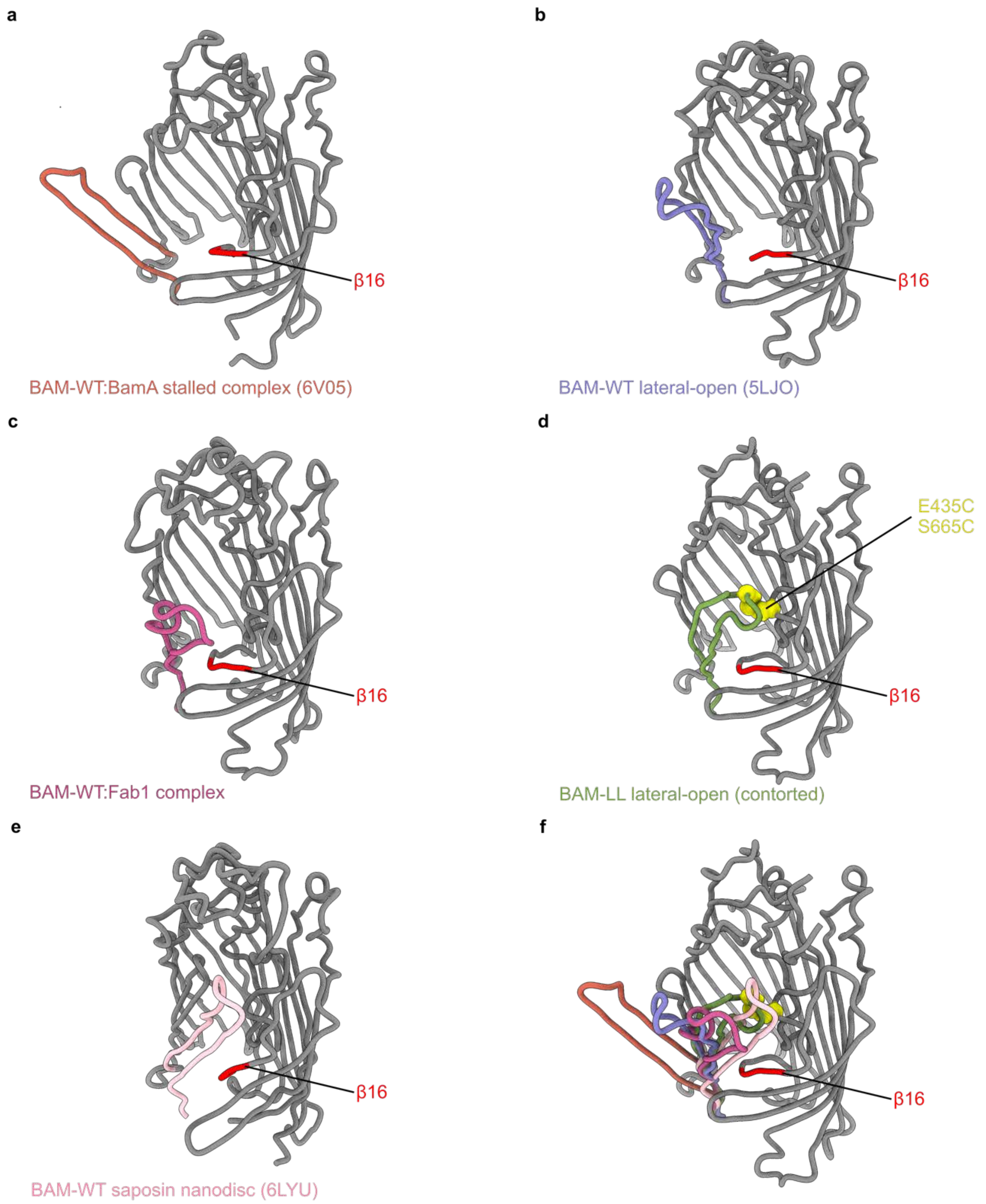

Supplementary Figure 9 | Conformational flexibility of BamA extracellular loop 1 in the lateral-open conformation. Atomic models of the BamA barrel are shown for several cryoEM structures of the BAM complex in lateral-open-like conformations: **(a)** a published structure of a substrate-engaged BAM complex (PDB ID 6V05[2]) **(b)** a published lateral-open structure of WT-BAM (PDB ID 5LJO[3]), **(c)** WT-BAM in complex with Fab1, **(d)** BAM-LL, and **(e)** a published structure of the BAM complex in a saposin nanodisc (PDB ID 6LYU[4]). Although in this nanodisc structure the lateral-gate is almost completely shut, we regard it as lateral-open-like as POTRA-5 still blocks the periplasmic side of BamA and has not moved out from under the barrel as seen in other lateral-closed structures. In addition, the BamA β-barrel width is narrowed, and the β1- β2 loop is bent into the barrel, as is seen in

LL-BAM. **(f)** Superposition of the conformation of β1-eL1-β2 for each structure, onto the full atomic model for the lateral-open lid-locked BAM complex, demonstrating the conformational variability of this feature. For each structure, β1, eL1 and β2 are highlighted, as well as β16 and the lid-lock disulphide (E435C S665C) where appropriate. Figure made in UCSF ChimeraX[5].

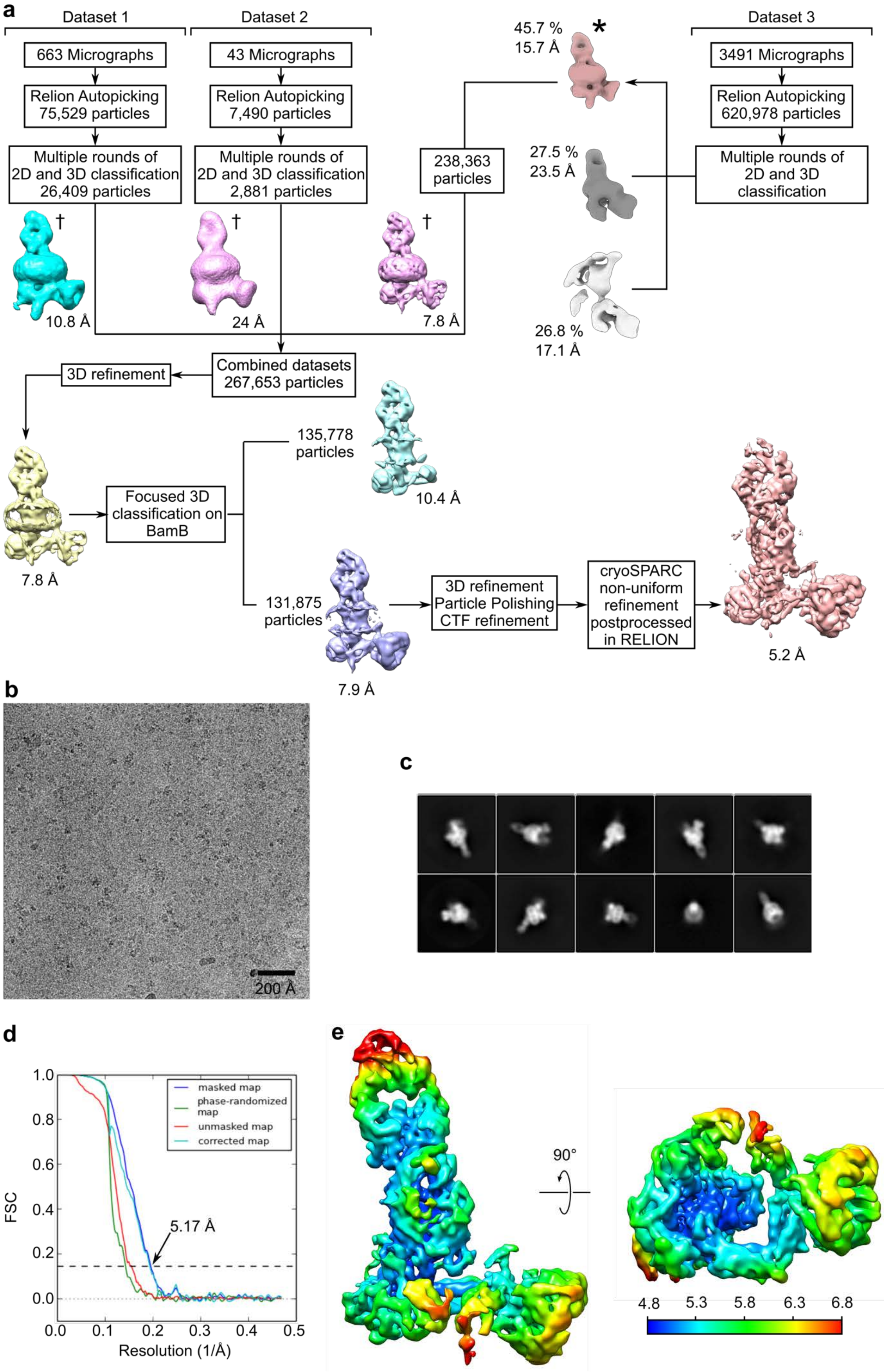
a
Dataset 1
Dataset 2
Dataset 3
663 Micrographs
43 Micrographs
3491 Micrographs
Relion Autopicking 75,529 particles
Relion Autopicking 7,490 particles
Relion Autopicking 620,978 particles
Multiple rounds of 2D and 3D classification 26,409 particles
Multiple rounds of 2D and 3D classification 2,881 particles
Multiple rounds of 2D and 3D classification
45.7 % 15.7 Å
27.5 % 23.5 Å
26.8 % 17.1 Å
238,363 particles
10.8 Å
24 Å
7.8 Å
Combined datasets 267,653 particles
3D refinement
7.8 Å
Focused 3D classification on BamB
135,778 particles
10.4 Å
131,875 particles
7.9 Å
3D refinement Particle Polishing CTF refinement
cryoSPARC non-uniform refinement postprocessed in RELION
5.2 Å
b
200 Å
c
d
FSC
Resolution (1/Å)
masked map
phase-randomized map
unmasked map
corrected map
5.17 Å
e
90°
4.8 5.3 5.8 6.3 6.8

Supplementary Figure 10 | Solving the structure of the BAM-Fab1 complex. (a) Image processing workflow for the BAM-WT-Fab1 complex in DDM detergent. The datasets were first processed individually through rounds of 2D and 3D classification. The individual class averages from the final 3D classification for dataset 3 are shown, the highest resolution of which (*) was taken forward. Similar results were obtained for the other datasets and the structures resulting from 3D refinement of the final particle stack for each are shown (†). Note at no point during processing of any dataset was a class average consistent with a lateral-closed state identified. Datasets were then combined, before running a final, focused classification to remove particles lacking BamB from the particle stack. Particles were then polished and CTF refined, before non-uniform refinement in cryoSPARC to generate the final structure. The structure determination process was performed once. **(b)** Representative micrograph and **(c)** representative 2D classes from dataset 1 (374.5 Å box). **(d)** FSC plot, calculated in RELION, used to estimate global resolution for the final reconstruction. **(e)** Final reconstruction, filtered and coloured by local-resolution as calculated in RELION. Map contour is 10.5 σ.

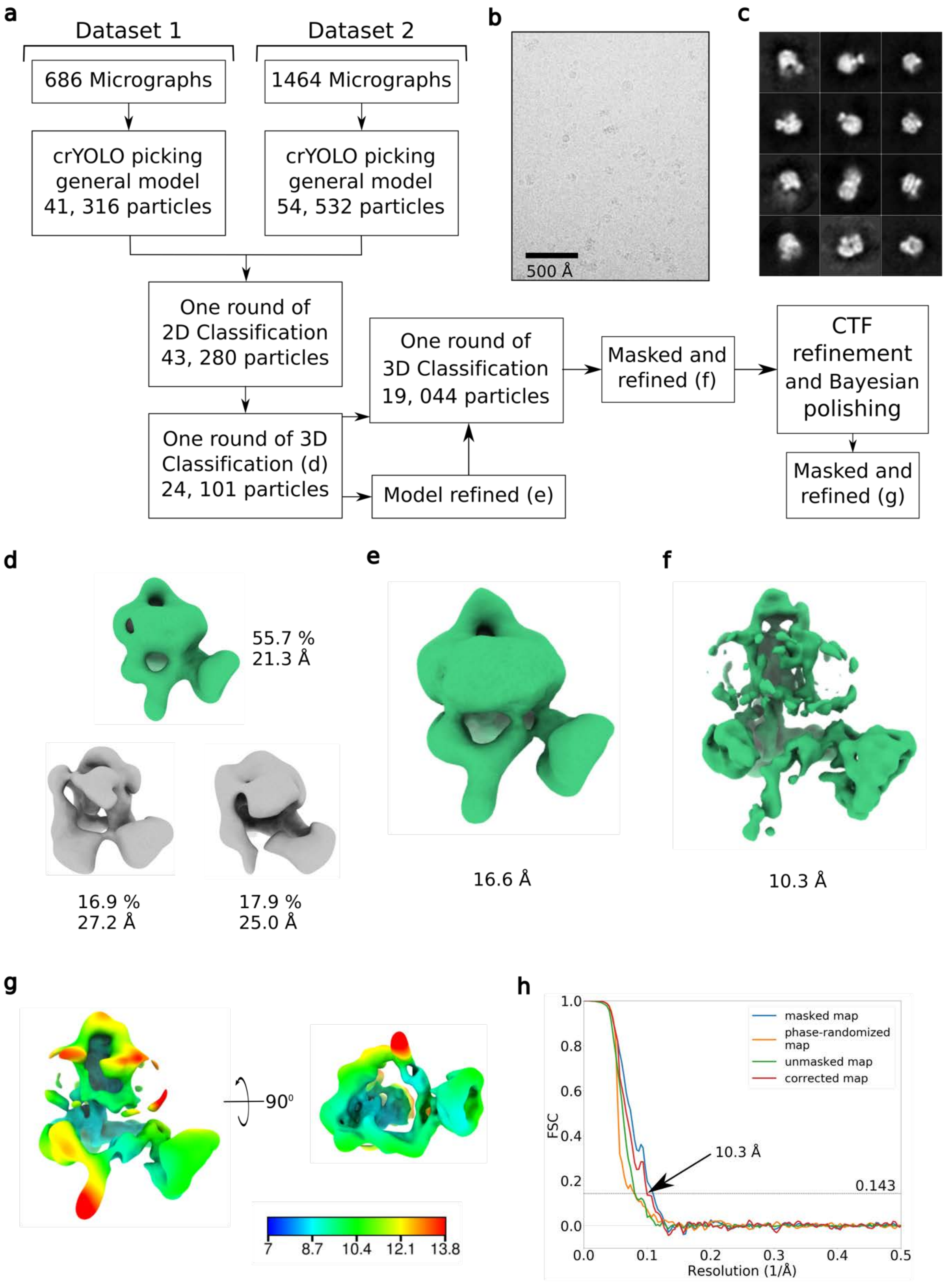

Supplementary Figure 11 | Solving the structure of BAM-P5L. (a) Image processing workflow for BAM-P5L in DDM detergent. Micrographs from dataset 1 and 2 were extracted into the same sized box, then pooled and processed together. **(b)** Representative micrograph from dataset 1. **(c)**

Representative 2D classes (300 Å box) from pooled dataset. The structure determination process was performed once. **(d)** Classes from the first 3D classification step, the particles in the green model were taken forward (map contour: 6 σ), the two grey classes were excluded (both map contours: 5 σ); there was also a fourth class of 10.3 % of the particles which didn't contain coherent density. **(e)** The initial refined model (contour: 6 σ), and **(f)** the model following further refinement and masking (contour: 8 σ). **(g)** FSC plot, calculated in RELION, used to estimate global resolution for the final model. **(h)** Final reconstruction filtered and coloured by local-resolution calculated in RELION (contour: 7.7 σ).

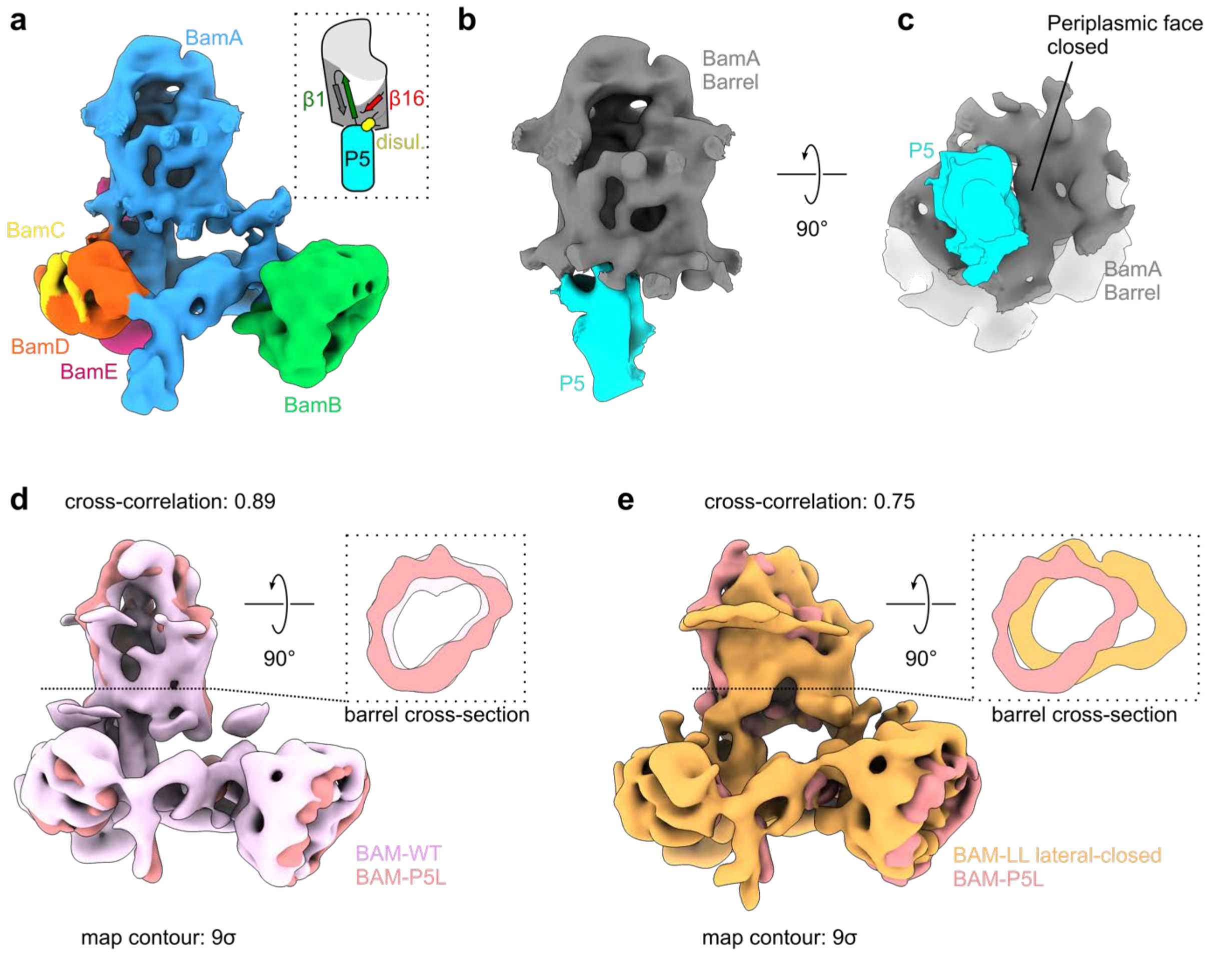

Supplementary Figure 12 | The BAM-P5L adopts a lateral-open conformation in DDM micelles. **(a)** 10.3 Å cryoEM map of BAM-P5L at a contour of 7 σ, coloured by subunit. Although atomic detail is not resolvable at this resolution, the position of POTRA-5 underneath the BamA barrel, as well as the overall barrel shape are consistent with a lateral-open conformation (*schematic inset*). Segmented density for the BamA barrel and POTRA-5 are shown viewed from **(b)** the barrel front, facing the lateral-gate, and **(c)** the periplasmic side of the barrel, showing that the BamA lumen is blocked by POTRA-5 in this conformation. **(d)** Alignment of BAM-P5L cryoEM density to WT BAM (EMDB-4061[3]), with calculated cross-correlation of the two maps shown above. Inset is a cross-section to compare the shape of the BamA barrel. The conformations are very similar both in terms of global fit, and the BamA barrel conformation. **(e)** Alignment of BAM-P5L cryoEM density to the BAM-LL lateral-closed conformation. The lower calculated cross-correlation between these two maps compared with (d), combined with the different barrel cross sections (shown inset), indicate BAM-P5L is not lateral-closed. For comparisons, BAM-WT and BAM-LL lateral-closed maps were lowpass filtered to the same resolution as BAM-P5L. Barrel cross-sections are shown at lower contour to maximise connectivity of the barrel density. Figure made in UCSF ChimeraX[5].

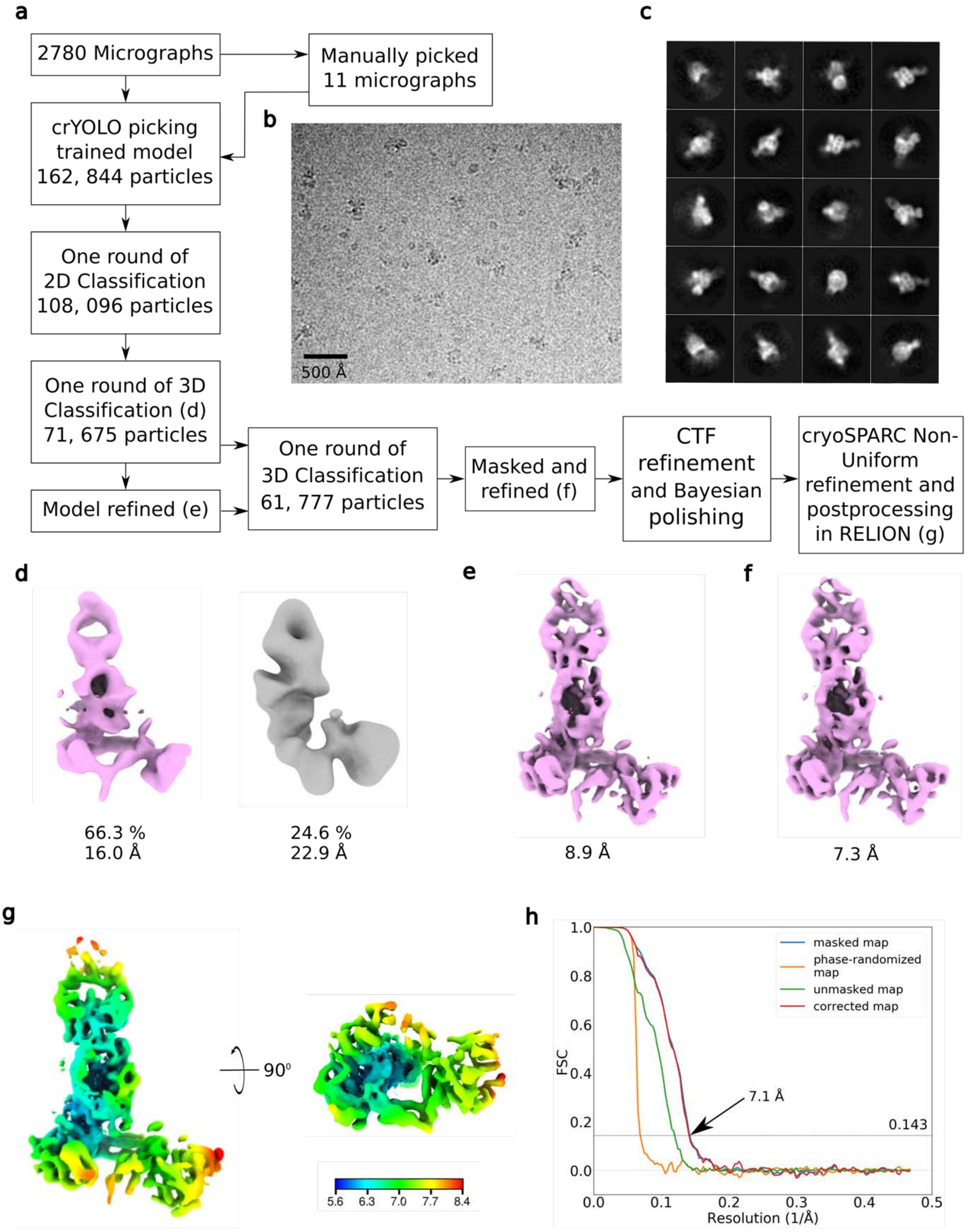

Supplementary Figure 13 | Solving the structure of BAM-LL:Fab1. (a) Image processing workflow for BAM-LL:Fab1 in DDM detergent. **(b)** Representative micrograph and **(c)** Representative 2D classes (321 Å box). The structure determination process was performed once. **(d)** Classification classes from first 3D classification step, the particles in the purple model were taken forward (map contour: 8 σ), particles in the grey class were excluded (map contour: 5 σ); there was a third class of 9.1 % of the particles which didn't contain coherent density. **(e)** The initial refined model (contour: 9

σ), and **(f)** the model following further refinement and masking (contour: 9.5 σ). **(g)** Final reconstruction following cryoSPARC non-uniform refinement, filtered and coloured by local-resolution calculated in RELION (contour: 9 σ). (**h**) FSC plot, calculated in RELION, used to estimate global resolution for the final model.

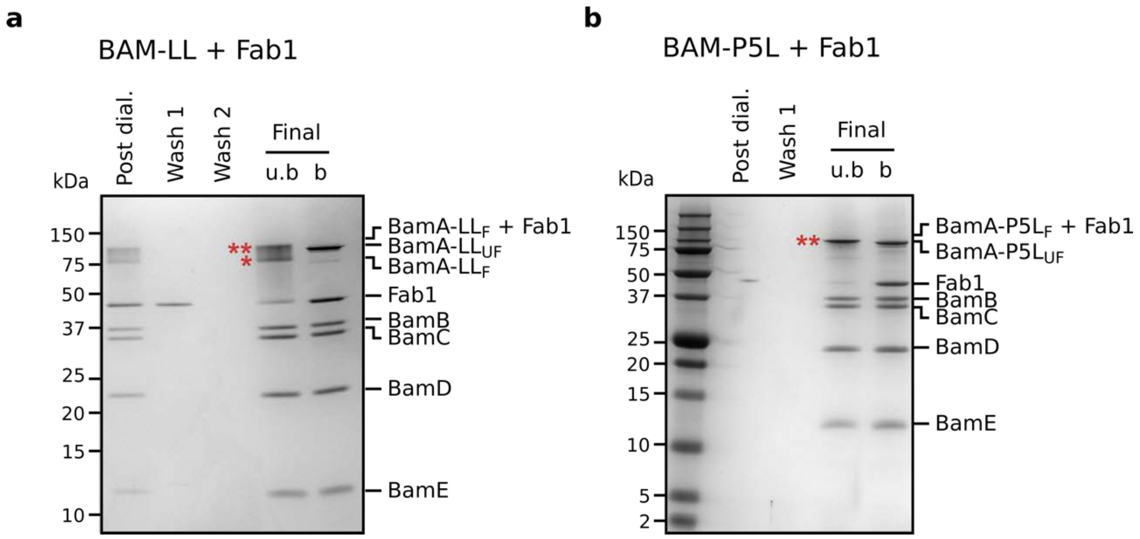

Supplementary Figure 14 | SDS-PAGE analysis of Fab1-bound BAM-LL and BAM-P5L *E. coli* polar lipid proteoliposomes. Quality of *E. coli* polar lipid proteoliposomes containing **(a)** Fab1-bound BAM-LL, and **(b)** Fab1-bound BAM-P5L assessed by SDS-PAGE. Samples of proteoliposomes immediately following dialysis (*post dial.*) along with samples of the wash (*see Methods*) were loaded to check successful reconstitution. 15 µL samples were mixed with 5 µL SDS-PAGE sample buffer and 15 µL was loaded on the gel. Samples were loaded boiled and un-boiled (*b and u.b,* respectively). Boiled samples were heated at > 95 °C for 10 min prior to loading. All five BAM subunits, along with Fab1 were present. The SDS-resistant complex with Fab1 (**) appears to be less stable for LL-BamA than for P5L-BamA, with a corresponding increase in non-complexed LL-BamA (*). These observations are consistent with Fab1 preferentially binding the lateral-open BamA conformation. This experiment was performed once for every proteoliposome variant produced for this study. Source data for all figure parts are provided as a source data file.

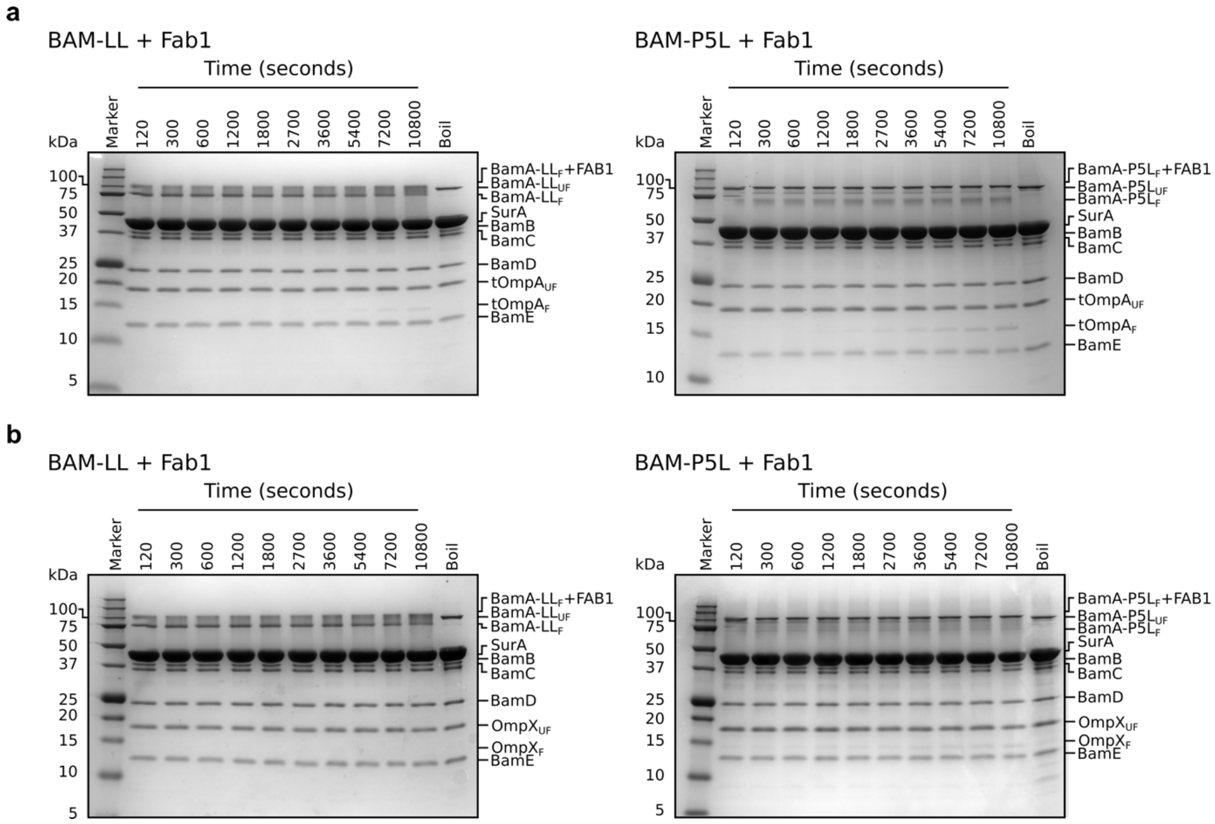

Supplementary Figure 15 | SDS-PAGE band-shift folding assays for Fab1-bound BAM-LL and BAM-P5L. Folding of **(a)** tOmpA and **(b)** OmpX by Fab1-bound BAM-LL and BAM-P5L variants was assayed by SDS-PAGE band-shift as described previously[28] where folded/unfolded species have different electrophoretic mobilities. Folding reactions contained 2 µM tOmpA or OmpX, 10 µM SurA and 1 µM BAM-containing *E. coli* polar lipid proteoliposomes in TBS pH 8.0, 0.8 M urea. Reactions were incubated at 25 °C and samples for SDS-PAGE analysis were taken at time intervals that were subsequently analysed on 15% (*w/v*) Tris-tricine SDS-PAGE gels at room temperature. A fully unfolded control (*Boil*) was included by boiling for 10 min at 95-100 °C prior to loading. Band intensities for folded and unfolded tOmpA (tOmpA_{F} and tOmpA_{UF}, respectively), and OmpX (OmpX_{F} and OmpX_{UF}, respectively) were quantified to determine the fraction folded at a given time point. Each experiment was repeated at least twice to confirm reproducibility. The number of replicates is shown in Supplementary Table 1. Source data for all figure parts are provided as a source data file.

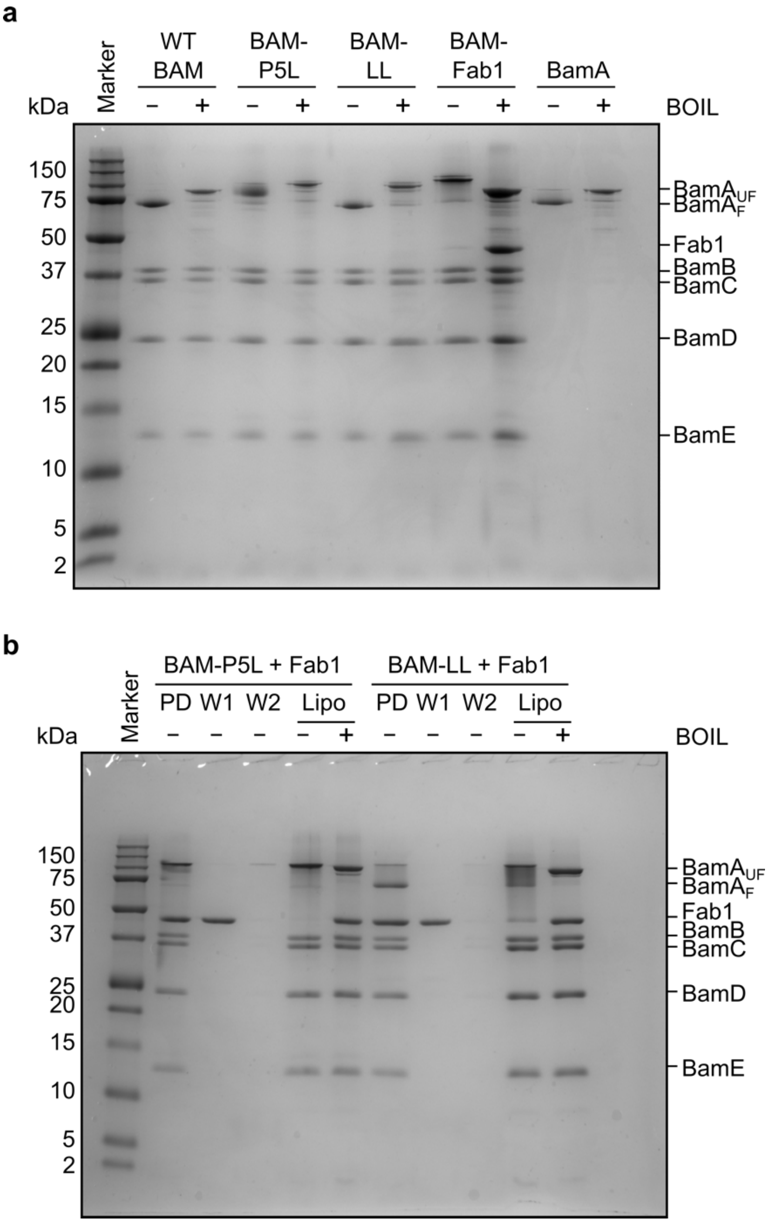

Supplementary Figure 16 | SDS-PAGE analysis of BAM/BamA-containing DMPC proteoliposomes. BAM/BamA-containing DMPC proteoliposomes were made at an LPR of 1600:1 (mol/mol) by dialysis (*see Methods*) and the quality of the proteoliposomes was analysed by SDS-PAGE. All five BAM subunits (and Fab1 for BAM-Fab1 complex proteoliposomes) were present and a mobility shift between boiled and un-boiled samples was observed, indicative of correctly folded BamA. **(a)** SDS-PAGE of DMPC proteoliposomes containing WT BAM, BAM-P5L, BAM-LL, BAM-Fab1 or BamA. **(b)** SDS-PAGE of DMPC proteoliposomes containing BAM-P5L + Fab1 or BAM-LL + Fab1 (*Lipo*), along with post-dialysis (*PD*) and two wash samples (*W1* and *W2*). Note that there are several mobility shifts observed for BamA due to differential migration of folded vs unfolded forms, the presence of a disulphide bond for BAM-P5L and BAM-LL, and the binding of Fab1. Boiled samples, denoted by (+), were heated to > 95 °C for 10 min prior to loading. 15 µL samples were mixed with 5 µL SDS-PAGE sample buffer and 15 µL was loaded on a 15% (*w/v*) Tris-tricine SDS-PAGE gel. This

experiment was performed once for every proteoliposome variant produced for this study. Source data for all figure parts are provided as a source data file.

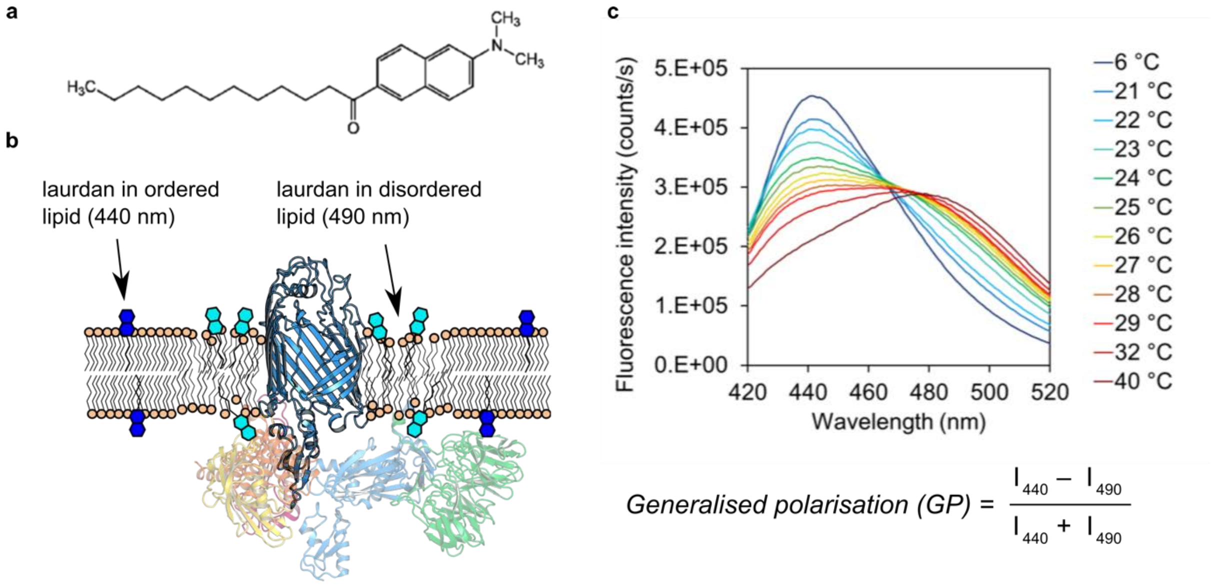

Supplementary Figure 17 | Probing lipid disorder using laurdan. (a) Chemical structure of laurdan. **(b)** All BAM complex variants, including BamA only and no protein controls were reconstituted into DMPC liposomes at an LPR of 1600:1 (*mol/mol*) and a final protein concentration of 0.8 µM, and laurdan was added to a final concentration of 4.2 µM. This equates to approximately 1 laurdan molecule for every 305 lipids and 5.25 laurdan per BAM complex. Laurdan incorporates into the liposomes stochastically and reports on the local order of the lipid environment. **(c)** Temperature dependence of fluorescence emission for laurdan-containing BAM-DMPC proteoliposomes under 340 nm excitation showing a shift in fluorescence emission maxima from 440 nm at 6 °C to 490 nm at 40 °C. The ratio of these maxima can be calculated as Generalised Polarisation (GP). Source data are provided as a source data file.

References

1. Kleinschmidt, J. H. Folding of β-barrel membrane proteins in lipid bilayers — Unassisted and assisted folding and insertion. *Biochim. Biophys. Acta - Biomembr.* **1848**, 1927–1943 (2015).
2. Tomasek, D. *et al.* Structure of a nascent membrane protein as it folds on the BAM complex. *Nature* **583**, 473–478 (2020).
3. Iadanza, M. G. *et al.* Lateral opening in the intact β-barrel assembly machinery captured by cryo-EM. *Nat. Commun.* **7**, 12865 (2016).
4. Xiao, L. *et al.* Structures of the β-barrel assembly machine recognizing outer membrane protein substrates. *FASEB J.* **35**, e21207 (2021).
5. Goddard, T. D. *et al.* UCSF ChimeraX: Meeting modern challenges in visualization and analysis. *Protein Sci.* **27**, 14–25 (2018).
