# Supplementary material for: The role of membrane destabilisation and protein dynamics in BAM catalysed OMP folding
Source: Nat Commun. 2021 Jul 7;12:4174. doi: 10.1038/s41467-021-24432-x (PMC8263589; doi:10.1038/s41467-021-24432-x)
Supplement: Supplementary file 2 — Reporting Summary [file 41467_2021_24432_MOESM2_ESM.pdf]

nature research

Corresponding author(s): Sheena Radford and Neil Ranson

Last updated by author(s): 21/5/21

Reporting Summary

Nature Research wishes to improve the reproducibility of the work that we publish. This form provides structure for consistency and transparency in reporting. For further information on Nature Research policies, see our Editorial Policies and the Editorial Policy Checklist.

Statistics

For all statistical analyses, confirm that the following items are present in the figure legend, table legend, main text, or Methods section.

n/a	Confirmed	
☐	☒	The exact sample size (*n*) for each experimental group/condition, given as a discrete number and unit of measurement
☐	☒	A statement on whether measurements were taken from distinct samples or whether the same sample was measured repeatedly
☒	☐	The statistical test(s) used AND whether they are one- or two-sided<br>*Only common tests should be described solely by name; describe more complex techniques in the Methods section.*
☒	☐	A description of all covariates tested
☒	☐	A description of any assumptions or corrections, such as tests of normality and adjustment for multiple comparisons
☐	☒	A full description of the statistical parameters including central tendency (e.g. means) or other basic estimates (e.g. regression coefficient) AND variation (e.g. standard deviation) or associated estimates of uncertainty (e.g. confidence intervals)
☒	☐	For null hypothesis testing, the test statistic (e.g. *F*, *t*, *r*) with confidence intervals, effect sizes, degrees of freedom and *P* value noted<br>*Give P values as exact values whenever suitable.*
☒	☐	For Bayesian analysis, information on the choice of priors and Markov chain Monte Carlo settings
☒	☐	For hierarchical and complex designs, identification of the appropriate level for tests and full reporting of outcomes
☒	☐	Estimates of effect sizes (e.g. Cohen's *d*, Pearson's *r*), indicating how they were calculated

Our web collection on statistics for biologists contains articles on many of the points above.

Software and code

Policy information about availability of computer code

Data collection	Electron microscopy data collected on a 300 keV Titan Krios at the Astbury Biostructure Laboratory using EPU software 1.8. Crystallographic data collected at Diamond Light Source Beamline i24.
Data analysis	Data were analyzed in Igor Pro v8.04. Electron microscopy data processed in RELION v_3.0. and v 3,1 Crystallographic data processed using CCP4 v.7.0.078. Motioncorr v2 gCTF v1.06, Phenix v 1.17.1, Coot v0.9-pre

For manuscripts utilizing custom algorithms or software that are central to the research but not yet described in published literature, software must be made available to editors and reviewers. We strongly encourage code deposition in a community repository (e.g. GitHub). See the Nature Research guidelines for submitting code & software for further information.

Data

Policy information about availability of data

All manuscripts must include a data availability statement. This statement should provide the following information, where applicable:

- Accession codes, unique identifiers, or web links for publicly available datasets
- A list of figures that have associated raw data
- A description of any restrictions on data availability

TThe final density maps are deposited in the Electron Microscopy Data Bank (EMDB) under accession numbers EMD-12232 [https://www.ebi.ac.uk/pdbe/entry/emdb/EMD-12232] (BAM-LL lateral-closed), EMD-12262 [https://www.ebi.ac.uk/pdbe/entry/emdb/EMD-12262] (BAM-LL lateral-open), EMD-12272 [https://www.ebi.ac.uk/pdbe/entry/emdb/EMD-12272] (BAM-Fab1 complex), EMD-12263 [https://www.ebi.ac.uk/pdbe/entry/emdb/EMD-12263] (BAM-P5L) and EMD-12271 [https://www.ebi.ac.uk/pdbe/entry/emdb/EMD-12271] (BAM-LL:Fab1 complex). Final model coordinates have been deposited in the Protein Data Bank (PDB) under accession numbers 7BNQ [https://doi.org/10.2210/pdb7BNQ/pdb] (BAM-LL lateral-closed), 7NBX [https://doi.org/10.2210/pdb7NBX/pdb] (BAM-LL lateral-open), 7ND0 [https://doi.org/10.2210/pdb7ND0/pdb] (BAM-Fab1 complex) and 7NCS [https://doi.org/10.2210/pdb7NCS/pdb] (BAM-LL:Fab1 complex).

Raw micrographs for each dataset will be deposited in the Electron Microscopy Public Image Archive (EMPIAR). The crystal structure of Fab1 has been deposited in the PDB under accession number 7BM5 [https://doi.org/10.2210/pdb7BM5/pdb], and crystallographic data are available at https://doi.org/10.2210/pdb7BM5/pdb. Source data are provided with this paper and are also freely available at the University of Leeds Data Repository: https://doi.org/10.5518/835.

Field-specific reporting

Please select the one below that is the best fit for your research. If you are not sure, read the appropriate sections before making your selection.

☒ Life sciences ☐ Behavioural & social sciences ☐ Ecological, evolutionary & environmental sciences

For a reference copy of the document with all sections, see nature.com/documents/nr-reporting-summary-flat.pdf

Life sciences study design

All studies must disclose on these points even when the disclosure is negative.

Sample size	no statistical methods were used to determine sample size. Experiments were repeated to ensure reproducibility.
Data exclusions	No data excluded.
Replication	All measurements were repeated (at least once for reproducibility) and gave similar results
Randomization	This is not relevant to our study, because no grouping was needed.
Blinding	Investigators were not blinded to group allocation, as no grouping was needed for this study.

Reporting for specific materials, systems and methods

We require information from authors about some types of materials, experimental systems and methods used in many studies. Here, indicate whether each material, system or method listed is relevant to your study. If you are not sure if a list item applies to your research, read the appropriate section before selecting a response.

Materials & experimental systems

n/a	Involved in the study
☐	☒ Antibodies
☒	☐ Eukaryotic cell lines
☒	☐ Palaeontology and archaeology
☒	☐ Animals and other organisms
☒	☐ Human research participants
☒	☐ Clinical data
☒	☐ Dual use research of concern

Methods

n/a	Involved in the study
☒	☐ ChIP-seq
☒	☐ Flow cytometry
☒	☐ MRI-based neuroimaging

Antibodies

Antibodies used	Antibody fab fragment described in this study (Fab1) is not used as an experimental tool/method but is a subject of the study
Validation	*Describe the validation of each primary antibody for the species and application, noting any validation statements on the manufacturer's website, relevant citations, antibody profiles in online databases, or data provided in the manuscript.*
